# Far-infrared irradiation attenuates vessel contraction by activating SERCA2 through disruption of SERCA2 and PLN interaction

**DOI:** 10.1371/journal.pone.0339066

**Published:** 2025-12-17

**Authors:** Yun-Jin Hwang, So-Young Park, Jung-Hyun Park, Du-Hyong Cho

**Affiliations:** 1 Department of Pharmacology, Yeungnam University College of Medicine, Nam-gu, Daegu, South Korea; 2 Department of Physiology, Yeungnam University College of Medicine, Nam-gu, Daegu, South Korea; 3 Department of Molecular Medicine, Ewha Womans University College of Medicine, Gangseo-gu, Seoul, South Korea; Army Medical University, CHINA

## Abstract

Vascular smooth muscle cells (VSMCs) plays an important role in maintaining vascular function by responding to various vasoactive stimuli within blood vessels. Far-infrared (FIR) rays has been shown to possess a variety of physiological effects including vasodilation, while the underlying molecular mechanism remains elusive. Here, we explored the molecular mechanism by which FIR irradiation suppresses vascular contraction using rat VSMCs and aortas. FIR irradiation enhanced the transport of intracellular Ca^2+^ from the cytosol to the sarcoendoplasmic reticulum (SER) via activation of sarcoendoplasmic reticulum Ca^2+^-ATPase (SERCA), which accompanied a decrease in intracellular ATP levels. Pretreatment with thapsigargin (TG), a specific SERCA inhibitor, or knockdown of SERCA2 gene expression reversed FIR irradiation-induced translocation of Ca^2+^ into the SER. Notably, FIR irradiation promoted the dissociation of SERCA2 and phospholamban (PLN), an endogenous SERCA inhibitor, without altering their total protein expression levels. The array of effects elicited by FIR irradiation was not observed under hyperthermic conditions (39°C). Moreover, FIR irradiation, but not hyperthermal condition, decreased the phosphorylation of myosin light chain (MLC) at Ser19, which was restored by pretreatment with TG or the knockdown of SERCA2 gene expression. FIR irradiation attenuated phenylephrine-induced vessel contraction in endothelium-deprived rat aortas. Consistent with the *in vitro* results, the reduction in MLC phosphorylation caused by FIR irradiation was reversed following pretreatment with TG in isolated aortas. Additionally, FIR irradiation increased blood flow in the carotid arteries of mice. Collectively, these results suggest that FIR irradiation activates SERCA2 by promoting its dissociation from PLN, independent of hyperthermic effects. This activation lowers cytosolic Ca²⁺ and ATP levels, reducing MLC phosphorylation and vascular smooth muscle contraction. These findings provide scientific evidence for the therapeutic potential of FIR therapy in the treatment and prevention of arterial narrowing conditions such as pathological vasospasm, and peripheral artery disease.

## Introduction

Vascular smooth muscle cells (VSMCs) are integral components of the medial layer of blood vessel walls. These cells respond to various biochemical and mechanical stimuli, including hormones, cytokines, and shear stress, and are crucial in maintaining vascular integrity and homeostasis by regulating blood pressure and distribution [1–3]. Hypercontractility of VSMCs has been implicated in the pathogenesis of various vascular diseases, including hypertension, atherosclerosis, and vasospastic conditions [3–6]. In this regard, recent studies have identified VSMC hypercontraction as the primary mechanism behind vasospasms rather than endothelial dysfunction [5,6], which can lead to severe vascular disorders and complications such as coronary artery spasm and subarachnoid hemorrhage (SAH)-induced cerebral vasospasm [5,6].

Enhanced VSMC contraction is primarily due to the influx of Ca^2+^ into VSMCs [7]. The phosphorylation of the 20-kDa myosin light chain (MLC) has been identified as a crucial step in VSMC contraction [8,9]. Myosin light chain kinase (MLCK) is activated in a Ca^2+^/calmodulin-dependent manner, and upon activation, phosphorylates MLC at Ser19, which leads to VSMC contraction [10]. Therefore, under normal circumstances, the levels of intracellular Ca^2+^ are tightly regulated and partitioned into specific areas. Specifically, the concentration of Ca^2+^ in the cytosol is approximately 100 nM, while in intracellular organelles it is around 10 µM. On the other hand, the extracellular fluid contains around 1 mM of Ca^2+^ [11,12].

All eukaryotic cells express the sarcoendoplasmic reticulum Ca^2+^-ATPase (SERCA) that functions largely to maintain the low cytosolic Ca^2+^ concentration by transporting Ca^2+^ across the sarcoendoplasmic reticulum (SER) membranes [13,14]. As can be inferred from the name Ca^2+^-ATPase, SERCA hydrolyzes one molecule of ATP to transport two cytosolic Ca^2+^ to SER against the concentration gradient [13,14]. Currently, three genes located on three different chromosomes have been identified to encode SERCA (e.g., SERCA1, SERCA2, and SERCA3) [13,14]. Unlike plasma membrane Ca^2+^-ATPase, which is activated by binding calmodulin or acidic phospholipids and polyunsaturated fatty acids, or phosphorylation at serine/threonine residues [15], SERCA have a unique regulation mode on its activity through the reversible binding of the endogenous small peptide inhibitor phospholamban (PLN) or sarcolipin (SLN) to SERCA [13,16,17]. SERCA activity is inhibited when PLN or SLN binds to SERCA, and its activity is enhanced once PLN or SLN is released from it [13,16,17]. PLN is a small integral membrane protein composed of 52 amino acids and mainly expressed in cardiac and smooth muscles, whereas SLN, a homolog of PLN, is composed of 31 amino acids and found in skeletal and atrial muscles [13,16,17].

Far-infrared (FIR) rays are a subset of non-visible electromagnetic radiation, defined by the International Commission on Illumination as encompassing wavelengths between 3 and 1000 μm [18]. When living cells are exposed to FIR rays, the energy from the rays is absorbed and modifies the vibrational state of bonds in different molecules such as water, protein, and lipid through changing several vibrational modes including stretching, scissoring, and twisting [19]. In addition to this effect, it is postulated that FIR irradiation elicits so-called meso-structure effect by which charged groups at specific sites on the proteins are associated with water molecules, which affects the dielectric behavior of the whole molecular-assembly, ultimately leading to diverse physiological effects in the body [19]. A number of studies have documented the beneficial effects of FIR irradiation on cardiovascular diseases, such as congestive heart failure, hypertension, and atherosclerosis [20,21]. In particular, repeated FIR therapy has been shown to prevent atherosclerosis by decreasing oxidative stress in individuals with coronary risk factors [22]. Additionally, FIR irradiation has been found to enhance arteriovenous fistula access flow and patency in patients undergoing hemodialysis [23].

Although studies have indicated that FIR rays possess vasoprotective properties, the specific molecular mechanisms underlying this effect have not been completely elucidated. Hence, the current study was conducted to explore the molecular mechanism by which FIR irradiation inhibits VSMC contraction in rat VSMCs and isolated aortas.

## Materials and methods

### Materials

Dimethyl sulfoxide (DMSO) and phenylephrine (PE) were obtained from Sigma–Aldrich (St. Louis, MO, USA). Thapsigargin (TG) were purchased from Cayman Chemicals (Ann Arbor, MI, USA). respectively. Antibodies against PLN and p-MLC-Ser^19^ were obtained from Cell Signaling Technology (Beverly, MA, USA). Antibodies against MLCK, SERCA2, and MLC were obtained from Santa Cruz Biotechnology (Dallas, TX, USA). The antibody against glyceraldehyde-3-phosphate dehydrogenase (GAPDH) was acquired from AbFrontier (Seoul, South Korea). Dulbecco’s modified Eagle’s medium (DMEM) was obtained from Fisher Scientific (Ottawa, Canada). Dulbecco’s phosphate-buffered saline (DPBS), fetal bovine serum (FBS), penicillin and streptomycin antibiotics, trypsin–EDTA solution, and plasticware for cell culture were supplied by Gibco-BRL (Gaithersburg, MD, USA). All other chemicals used were of the purest analytical grade available.

### Cell culture and drug treatments

Rat aortic VSMCs were isolated and cultured as previously described [24]. Briefly, six-week old male Sprague-Dawley (SD) rats (KOATECH, Pyeongtaek-si, South Korea) were euthanized with CO_2_ gas and underwent cervical dislocation, and their thoracic aortas were immediately dissected. Connective tissues were removed and the endothelium was denuded by gentle rubbing. The endothelium-deprived aortas were cut into 1-mm pieces and grown in DMEM supplemented with 10% FBS at 37°C under 5% CO_2_ humidified air. Cells between passages 3–7 were used for all experiments. VSMCs grown to 90% confluence in 60-mm or 35-mm culture dishes were incubated in the absence or presence of FIR ray for the indicated times in DMEM supplemented with 2% FBS. In some experiments, VSMCs were treated with the indicated drugs or chemicals for the indicated times. In a separate study, cells were also incubated at 25°C (room temperature, RT) or 39°C on the heating block for 30 min.

### FIR irradiation

Rat VSMCs or isolated rat aortas were irradiated with FIR ray using a ceramic infrared radiator (Model No. IOT/90–250, Elstein-Werk M. Steinmetz GmbH & Co. KG, Northeim, Germany), as previously performed in our laboratory [25,26]. It was confirmed that the emission wavelength of the FIR radiator ranged from 1 to 20 μm with a 4 μm peak wave-length, and the irradiance at the surface of the FIR radiator and the place where a 60-mm culture dish was 2,530 mW/cm^2^ and 65 mW/cm^2^, respectively [25,26]. Using the FIR generator, VSMCs or rat aortas cultured in a 60-mm culture dish with 3 mL of the medium were exposed to FIR rays at RT for the indicated time (0, 15, 30, or 45 min). For hyperthermal control experiments, VSMCs in a 60-mm culture dish were placed on the heat block set at 39°C for 30 min.

### Western blot analyses

VSMCs irradiated with FIR ray in the absence or presence of various chemicals were lysed in lysis buffer [20 mM Tris-HCl pH 7.5, 150 mM NaCl, 1% Triton X-100, 1 mM EDTA, 1 mM EGTA, 1 mM PMSF, 10 mM β-glycerophosphate, 1 mM NaF, 1 mM Na_3_VO_4_, and 1 × protease inhibitor cocktail from Roche Molecular Biochemicals (Indianapolis, IN, USA)]. In addition to VSMCs, the endothelium-deprived rat aortas were either irradiated with FIR rays or not. The proteins were extracted by chopping the aortic tissues using iris scissors in an ice-cold lysis buffer as previously described [27]. The protein concentration was determined using a BCA protein assay kit from Thermo Fisher Scientific (Waltham, MA, USA). Equal quantities of protein (20 µg) were separated via sodium dodecyl sulfate-polyacrylamide gel electrophoresis with 8‒12% gels, transferred to a nitrocellulose (NC) membrane from Cytiva (Marlborough, MA, USA) and then blocked using 5% skim milk for 1 h at RT. NC membranes were incubated overnight with primary antibodies at 4˚C. After overnight, blots were probed with corresponding secondary antibodies, which were all obtained from Thermo Fisher Scientific (1:5,000), for 1 h at RT and finally developed using electrochemiluminescence reagents from Cytiva. Densitometry was performed to quantify the phosphorylation levels of proteins relative to corresponding total protein expressions or total protein expressions relative to GAPDH expressions using ImageJ (NIH, Bethesda, MD, USA). The primary antibody dilutions used for western blot analyses were as follows: SERCA2 (1:1,000), PLN (1:1,000), MLCK (1:5,000), p-MLC-Ser^19^ (1:1,000), MLC (1:1,000), and GAPDH (1:3,000).

### Detection of intracellular Ca^2+^ localization

Intracellular Ca^2+^ localization was detected using Fura-2 AM (Thermo Fisher Scientific), an intracellular Ca^2+^ indicator, and following the manufacturer’s protocol. Briefly, VSMCs grown on coverslips were incubated for 30 min in the absence or presence of 1 μM TG in DMEM supplemented with 2% FBS, followed by FIR irradiation for 30 min or not in the presence of 2 μM Fura-2 AM and 1 μM ER tracker (Thermo Fisher Scientific). The intracellular Ca^2+^ localization and ER location were immediately detected in living cells using a confocal microscope (K1-Fluo; Nanoscope Systems Inc., Daejeon, South Korea). The nuclei were also detected using 1 μM Hoechst33342 (Tocris Bioscience, Bristol, UK). The Ca² ⁺ levels in FIR-irradiated live cells were then quantified using ImageJ software (NIH). The merged signal (yellow), representing the co-localization of Ca²⁺ (green) and SER (red) signals, was divided by the total cellular Ca² ⁺ signal (green), and the value was expressed as a percentage.

### Measurement of intracellular ATP levels

Intracellular ATP levels were measured using a luminescent ATP detection assay kit (Cayman Chemicals, MI, USA) following the manufacturer’s instructions. Briefly, VSMCs were seeded into 35-mm culture dishes. When VSMCs reached 90% confluence, the cells were incubated for 30 min in the absence or presence of 1 μM TG in DMEM supplemented with 2% FBS, followed by FIR irradiation for 30 min or not. The cells were washed once with ice-cold DPBS, and the cell lysates were obtained using a 1 × ATP assay sample buffer (40 µL). The supernatants were collected by centrifugation, and an ATP reaction buffer (110 µL) containing D-luciferin and luciferase was added to 10 µL of each cell supernatant. The luminescence was then measured using a microplate reader (Molecular Devices, Sunnyvale, CA, USA). The final intracellular ATP levels were obtained by normalizing the luminescence values to their total protein contents.

### Immunofluorescence assay

Confocal microscopic analyses using cells grown on coverslips were performed as previously described [26]. Briefly, VSMCs were seeded on coverslips in 35-mm culture dishes. After irradiation without or with FIR ray for 30 min, cells were fixed in 4% (wt/vol) paraformaldehyde in DPBS and washed with 50 mM NH_4_Cl, followed by 5 min of permeabilization in 0.2% (vol/vol) Triton X-100 in DPBS at RT. After permeabilization, cells were blocked in 2% (wt/vol) bovine serum albumin in DPBS for 10 min. The presence of MLCK (1:500), p-MLC-Ser^19^ (1:100), SERCA2 (1:100) and PLN (1:100) was detected using the appropriate primary antibody, followed by the use of the Alexa Fluor 488- or 594-conjugated secondary antibody (Thermo Fisher Scientific). The nuclei were detected using 1 μM Hoechst33342 (Tocris Bioscience). Colocalized images were photographed using a confocal microscope (K1-Fluo; Nanoscope Systems Inc.).

### Co-immunoprecipitation (co-IP)

After irradiation with or without FIR rays for 30 min, VSMCs were lysed with lysis buffer B (20 mM Tris-HCl pH 7.5, 150 mM NaCl, 1% NP-40, 1 mM EDTA, 1 mM EGTA, 1 mM PMSF, 10 mM β-glycerophosphate, 1 mM NaF, 1 mM Na_3_VO_4_, and 1 × PIC) and centrifuged at 17,000 *g* for 15 min at 4°C. The supernatant (500 μg protein) was precleared with 40 μL of a 50% slurry of preequilibrated protein A/G-agarose beads (Thermo Fisher Scientific) at 4°C for 2 h. The precleared supernatant was incubated at 4°C for 16 h with 4 μL of the SERCA2 antibody or 4 μL of normal rabbit IgG, and then for 2 h at 4°C with 40 μL of a 50% slurry of preequilibrated protein A/G-agarose beads. The immunoprecipitates were washed thoroughly three times with lysis buffer B. The bound proteins were eluted with 20 μL of 1 × Laemmli sample buffer and subjected to western blot analysis using the appropriate antibodies.

### Knockdown of SERCA2 gene expression

The knockdown of SERCA2 gene expression using siRNA was performed as previously described [28]. Briefly, VSMCs grown to 70% confluence in 35-mm culture dishes were transfected with 100 nM of rat *Atp2a2* (SERCA2) siRNA (Dharmacon Inc., Lafayette, CO, USA) or 100 nM of scramble siRNA (Dharmacon Inc.) using DharmaFECT2 (Dharmacon Inc.). After incubation for 5 h at 37ºC, the DharmaFECT mixtures were washed out, and cells were further incubated in DMEM supplemented with 10% FBS for 24 h before exposure to FIR rays.

### SERCA activity assay

Ca^2+^-dependent SERCA activity was measured in cell lysates through a spectrophotometric assay using an enzyme-coupled system as previously described [29]. Briefly, after exposure to FIR rays or not for 30 min, cells were homogenized with homogenization buffer (25 mM digitonin, 250 mM sucrose, 5 mM HEPES pH 7.0, 1 mM PMSF, and 1 × PIC). Protein concentration was adjusted with the homogenization buffer to 60 μg/mL and added into the reaction buffer (100 mM KCl, 10 mM MgCl_2_, 20 mM HEPES, pH 7.0, 10 mM phosphoenolpyruvate, 1 mM EGTA, 15 U/mL of each of pyruvate kinase and lactate dehydrogenase, 0.5 mM NADH, and 5 mM free Ca^2+^). The reactions were initiated by adding 5 mM ATP, and the decrease in absorbance at 340 nm was recorded using a spectrophotometer (GENESYS 10; Thermo Fisher Scientific) at 37°C for 10 min. The SERCA-independent Ca^2+^-ATPase activity was measured in the presence of the SERCA inhibitor TG (10 μM) and subtracted.

### Fluorescence resonance energy transfer (FRET) assay

Human PLN cDNA (Origene, Rockville, MD, USA) and human SERCA2a cDNA (Addgene, Watertown, MA, USA) were subcloned into a pcDNA3 vector containing the cyan fluorescent protein (CFP) gene (Addgene) and the yellow fluorescent protein (YFP) gene (Addgene), respectively. HEK293T cells (ATCC, Manassas, VA, USA) grown to 70% confluence in 12 well-culture plates in DMEM supplemented with 10% FBS were co-transfected with 0.5 μg of the CFP-tagged PLN DNA construct and 0.5 μg of the YFP-tagged SERCA2a DNA construct using Lipofectamine 2000 (Invitrogen, Carlsbad, CA, USA), according to the manufacturer’s instructions. After incubation for 5 h at 37°C, the culture medium was washed out, and the cells were further incubated in DMEM supplemented with 10% FBS for 24 h before exposure to FIR rays. On the day the FRET assay was performed, transfected cells were incubated for 30 min at 39°C using the heat block or for 30 min at RT, or were exposed to FIR ray for 30 min at RT. The fluorescence was measured using a microplate reader (SpectraMax iD5; Molecular Devices) with an excitation wavelength of 480 nm and an emission wavelength of 530 nm. The final FRET efficiency was obtained by normalizing the fluorescence values at 530 nm to their total protein contents.

### Animals

All animal experiments were conducted in accordance with the approved institutional guidelines for animal care and use at Yeungnam University (Approval Nos. YUMC-AEC2021–020 and YUMC-AEC2024–031). The protocol for these experiments was approved by the Institutional Animal Care and Use Committee of Yeungnam University. Additionally, all animal experiments performed in this study complied with the Animal Research Reporting In Vivo Experiments guidelines. Six-week-old male SD rats and seven-week-old male C57BL/6 mice (KOATECH) were maintained for a week at the beginning of the experiment in a temperature- and humidity-controlled room (22 ± 1°C and 50 ± 10%, respectively) under a 12-h alternate light/dark cycle. All rats were given water and fed a standard chow from Purina Mills, LLC (St. Louis, MO, USA) ad libitum throughout the experiments.

### Measurement of aortic vessel contraction

Aortic vessel contractions were measured in thoracic aortic rings as previously described [30] with minor modiﬁcations. Brieﬂy, the endothelium-deprived aorta was cut into 5-mm ring segments. The prepared aortic ring segments were incubated for 1 h in the absence or presence of 1 μM TG in DMEM supplemented with 2% FBS at 37°C under a 5% CO_2_ humidiﬁed air, and exposed to FIR ray or not for 30 min. The aortic rings were then mounted on L-shaped holders in 7 ml organ baths containing a warmed (37°C) and oxygenated (95% O_2_ and 5% CO_2_) Krebs-Henseleit (KH) solution (118.1 mM NaCl, 4.7 mM KCl, 2.6 mM CaCl_2_, 0.6 mM MgSO_4_, 24.9 mM NaHCO_3_, 1.2 mM KH_2_PO_4_, and 5.6 mM glucose). The muscle force was recorded isometrically through a force transducer (MP35) connected to a BLS analyses software, all from BIOPAC Systems Inc. (Goleta, CA, USA). The rings were stretched to a resting tension of 2 g and equilibrated for 30 min in an organ bath ﬁlled with KH solution. The rings were then sequentially exposed to 65 mM KCl and the KH solution at least twice, and PE (0.1‒100 μM) was cumulatively added to determine aortic vessel contraction.

### Ultrasound imaging

Peripheral blood flow to the skin vasculature was assessed by measuring the blood flow rate in the carotid artery of mice using ultrasound imaging analysis. Anesthetization of the mice was induced with 4% isoflurane (Hana Pharm Co, Seoul, South Korea) and maintained with 1–2% isoflurane. The mice were then placed on the heated animal imaging platform. The hair in the neck region was removed using a depilatory cream, and the ultrasound MS550D transducer (Fujifilm VisualSonics, Toronto, ON, Canada) was placed perpendicularly to the mouse’s neck. The ultrasound images of the mouse carotid artery were acquired using the MS550D transducer at an operating frequency of 32–56 MHz. After acquiring control ultrasound images, the mouse was exposed to FIR ray for 20 min under the same irradiation conditions as those in the *in vitro* experiments, followed by the acquisition of ultrasound images. Vessel diameter and blood flow velocity were measured in the carotid artery of mice using color Doppler mode and pulsed-wave Doppler mode, respectively, with the Vevo 2100 system (Fujifilm VisualSonics, Toronto, ON, Canada). The blood flow rate was calculated by multiplying the vessel diameter by the mean blood flow velocity.

### Statistical analyses

All results except PE-induced aortic vessel contraction results are expressed as mean ± standard deviation (SD) values, with n indicating the number of experiments. PE-induced aortic vessel contraction data are expressed as the mean ± standard error (SE) at each point. The statistical significance of differences between two mean values was assessed using Student’s *t*-test, whereas differences among more than two mean values were evaluated with two-way analysis of variance (ANOVA) followed by Tukey’s *post hoc* test, using GraphPad Prism (GraphPad Software, San Diego, CA, USA) for analysis. All differences were considered significant at a *P* value < 0.05.

## Results

### FIR irradiation promotes the movement of cytosolic Ca^2+^ into the SER by stimulating SERCA2 activity in VSMCs

**Fig 1:**
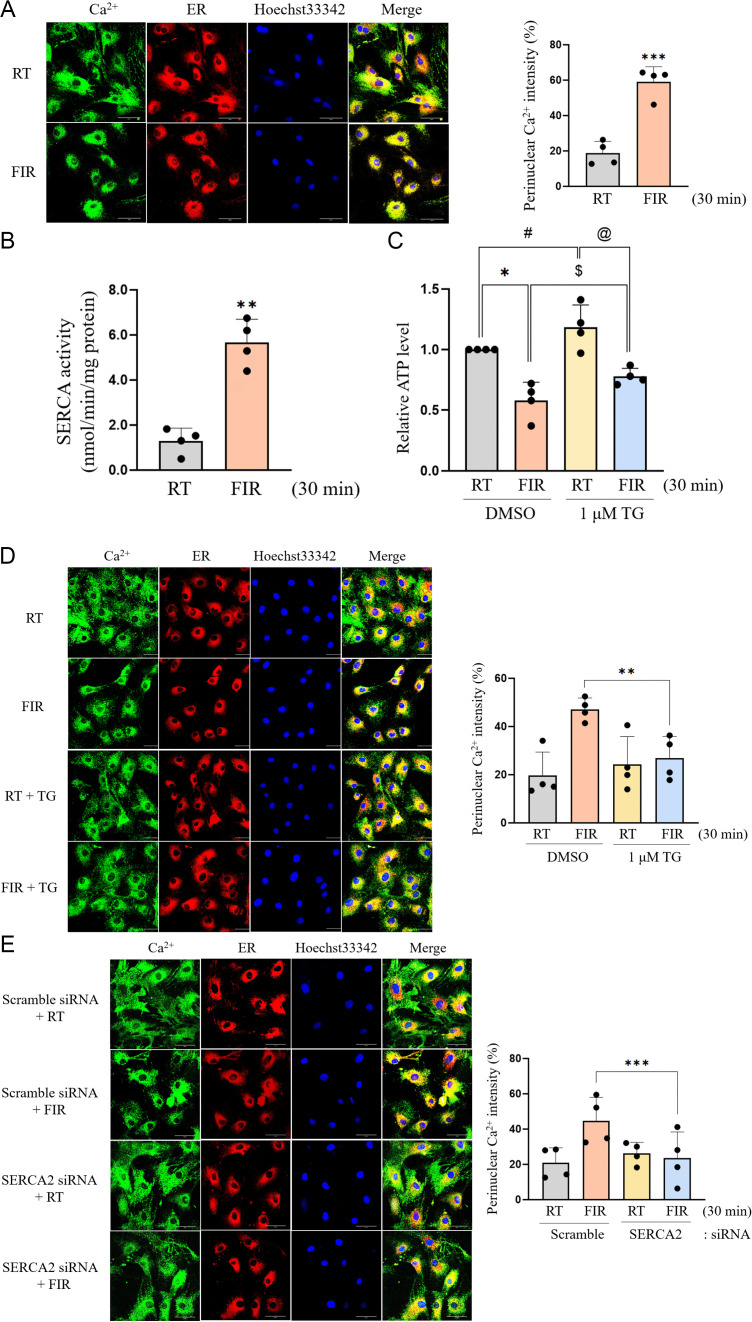
Intracellular Ca^2+^ is translocated from the cytosol to the SER by FIR irradiation-activated SERCA2 in VSMCs. (A) VSMCs grown on coverslips were irradiated with FIR ray or not for 30 min in the presence of 2 μM Fura-2 AM and 1 μM ER tracker. Subcellular Ca^2+^ locations were detected in Materials and Methods. The nuclei were also detected using 1 μM Hoechst33342. The scale bar indicates 50 µm. (B) VSMCs were exposed to FIR ray or not for 30 min and then Ca^2+^-dependent SERCA activity in cell lysate was assessed by a spectrophotometric assay using an enzyme-coupled system as described in Materials and Methods. SERCA-independent Ca^2+^-ATPase activity was also measured in the presence of the SERCA inhibitor TG (10 μM) and subtracted. (C) VSMCs grown to 90% confluence in 35-mm culture dishes were incubated for 30 min in the absence or presence of 1 μM TG, irradiated with FIR ray or not for 30 min and then intracellular ATP levels were measured using a luminescent ATP detection assay kit as described in Materials and Methods. (D) VSMCs grown on coverslips were incubated for 30 min in the absence or presence of 1 μM TG in DMEM supplemented with 2% FBS, followed by FIR irradiation for 30 min or not in the presence of 2 μM Fura-2 AM and 1 μM ER tracker. Subcellular Ca^2+^ locations were detected as described in Materials and Methods. The nuclei were also detected using 1 μM Hoechst33342. The scale bar indicates 50 µm. (E) VSMCs grown on coverslips were transfected with SERCA2-specific siRNA or scramble siRNA, followed by FIR irradiation for 30 min or not in the presence of 2 μM Fura-2 AM and 1 μM ER tracker. Subcellular Ca^2+^ locations were detected as described in Materials and Methods. The nuclei were also detected using 1 μM Hoechst33342. The scale bar indicates 50 µm. All experiments were performed at least four times independently, and the confocal microscopic photographs shown are representative of at least four experiments (n = 4). The scale bar indicates 30 µm. Bar graphs depict mean fold alterations above/below the controls (± SD). Differences were considered to be statistically significant at ^*^P < 0.05, ^#^*P* < 0.05, ^$^*P* < 0.05, ^@^*P* < 0.05, ^**^P < 0.01, and ^***^P < 0.001.

It has been recently reported that FIR irradiation changes subcellular Ca^2+^ localization from the cytosol into the nucleus in MDA-MB-231 human breast cancer cells [26]. Therefore, we first examined whether FIR irradiation also alters intracellular Ca^2+^ localization in VSMCs. Intracellular Ca^2+^ was localized to the perinucleus when cells were exposed to FIR ray for 30 min, compared to room temperature (RT) control in which intracellular Ca^2+^ was diffusely detected in the cytosol (S1 Fig). To further clarify the Ca^2+^ location in FIR-irradiated cells, confocal microscopic analyses were performed using an ER tracker, and it was observed that FIR irradiation for 30 min almost completely localized intracellular Ca^2+^ to the SER (Fig 1A). It is well-known that SERCA is the only Ca^2+^-ATPase pump involved in the transport of cytosolic Ca^2+^ into the SER [31]. Therefore, we examined whether FIR irradiation enhances SERCA activity in VSMCs. As expected, FIR irradiation for 30 min increased SERCA activity by 4.5-fold, compared to that in the RT control (Fig 1B). Furthermore, intracellular ATP levels in FIR-irradiated VSMCs were decreased to 40% of those in the RT control, and pretreatment with 1 μM TG, a specific SERCA inhibitor [32], significantly restored the FIR irradiation-decreased intracellular ATP levels (Fig 1C), suggesting that FIR irradiation-activated SERCA consumed large amounts of intracellular ATP to transport cytosolic Ca^2+^ into the SER. Next, we tested whether FIR irradiation-activated SERCA substantially mediates this Ca^2+^ transport. As shown in Fig 1D, intracellular Ca^2+^ localization to the SER by FIR irradiation was significantly reversed by pretreatment with TG. To clearly confirm the role of SERCA in Ca^2+^ transport to the SER by FIR irradiation, *Atp2a2* siRNA specific to the rat SERCA2 gene was introduced into VSMCs. The knockdown of SERCA2 gene expression was successful, as evidenced by decreased SERCA2 expression in *Atp2a2* siRNA-transfected VSMCs (S2 Fig). Similar to pharmacological inhibitor studies using TG, diffused cytosolic Ca^2+^ distribution was observed in SERCA2-knockdown cells even though FIR rays were irradiated for 30 min, whereas the SER localization of intracellular Ca^2+^ was observed in FIR-irradiated control cells (Fig 1E). Taken together, these findings indicated that FIR irradiation caused the translocation of cytosolic Ca^2+^ into the SER by activating SERCA2, which in turn consumed intracellular ATP.

### SERCA2 is activated by decreased binding of SERCA2 and PLN in FIR-irradiated VSMCs

**Fig 2:**
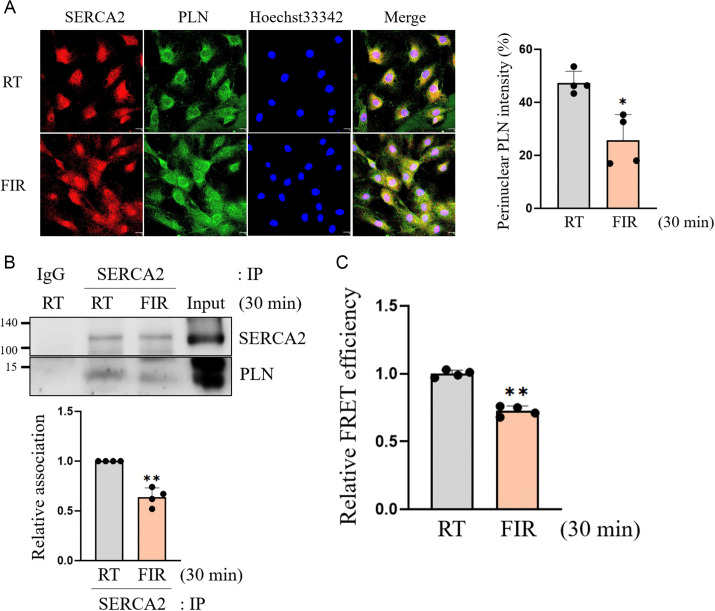
FIR irradiation activates SERCA2 by decreasing binding of SERCA2 and PLN in VSMCs. (A) VSMCs grown on coverslips were irradiated with FIR ray or not for 30 min, and the location of SERCA2 or PLN was detected by confocal microscopic analyses as described in Materials and Methods. The nuclei were also detected using 1 μM Hoechst33342. The scale bar indicates 30 µm. (B) VSMCs were irradiated with FIR ray or not for 30 min and total proteins were obtained, and then the co-IP assay for SERCA2 and PLN was performed using 500 μg of each protein as described in Materials and Methods. (C) HEK293T cells grown to 70% confluence in 12 well-culture plates were co-transfected with human CFP-tagged PLN plasmids and YFP-tagged SERCA2a plasmids, followed by irradiation with FIR ray or not for 30 min, and then the FRET values were measured as described in Materials and Methods. All experiments were performed at least four times independently, and the confocal microscopic photographs and blots shown are representative of at least four experiments (n = 4). The bar graph depicts mean fold alterations below the controls (± SD). Differences were considered statistically significant at *P < 0.05 and **P < 0.01.

It is well-known that PLN, an endogenous small peptide inhibitor [31], binds to SERCA, inhibiting of SERCA activity, and SERCA is activated when PLN is dissociated from it [31]. To unravel the molecular mechanism through which FIR irradiation activates SERCA2, we first tested whether FIR irradiation alters the expression of SERCA2 and PLN in VSMCs. However, no changes were observed in the expression of SERCA2 and PLN in FIR-irradiated VSMCs (S3 Fig). Although expression status of SERCA2 and PLN did not change in FIR-irradiated VSMCs, confocal microscopic results showed that FIR irradiation for 30 min remarkably decreased the colocalization of SERCA2 and PLN (Fig 2A). To confirm these confocal microscopic results, co-IP assay was performed, and the co-IP results clearly revealed that the physical association of SERCA2 and PLN in FIR-irradiated VSMCs decreased to 60% of that in the RT control (Fig 2B). To further confirm these quantitative results, FRET assay was conducted by co-transfecting CFP-tagged PLN and YFP-tagged SERCA2 into HEK293T cells. In accordance with the co-IP results, the FRET efficiency was significantly reduced by FIR irradiation (Fig 2C), indicating that FIR irradiation promoted the dissociation of SERCA2 and PLN. These results demonstrated that FIR irradiation enhanced SERCA2 activity by promoting the dissociation of SERCA2 and PLN.

### Peculiar action of FIR ray, not hyperthermal effect, induces SERCA2-mediated SER localization of intracellular Ca^2+^

**Fig 3:**
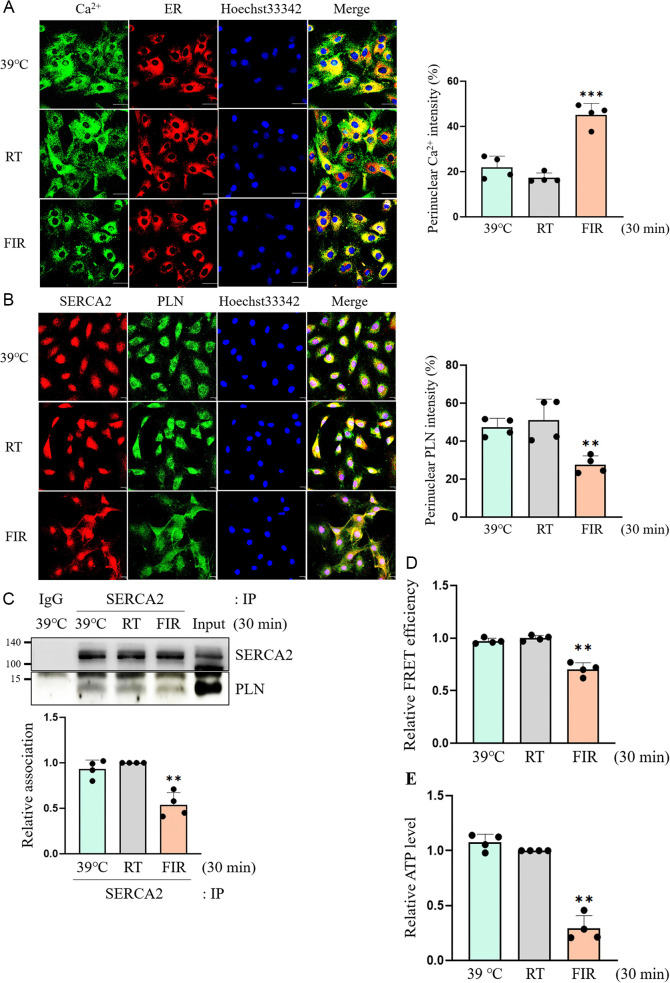
Distinctive effect of FIR ray, not hyperthermal effect, promotes SERCA2-mediated SER localization of intracellular Ca^2+^. (A) VSMCs grown on coverslips were incubated for 30 min at 39°C using the heat block or for 30 min at RT, or were exposed to FIR ray for 30 min at RT in the presence of 2 μM Fura-2 AM and 1 μM ER tracker, and then subcellular Ca^2+^ locations were detected as described in Materials and Methods. The nuclei were also detected using 1 μM Hoechst33342. The scale bar indicates 50 µm. (B) VSMCs grown on coverslips were incubated for 30 min at 39°C using the heat block, for 30 min at RT, or were exposed to FIR ray for 30 min at RT, and the location of SERCA2 and PLN was detected by confocal microscopic analyses as described in Materials and Methods. The nuclei were also detected using 1 μM Hoechst33342. The scale bar indicates 30 µm. (C) VSMCs were incubated for 30 min at 39°C using the heat block or for 30 min at RT or were exposed to FIR ray for 30 min at RT, and then the co-IP assay for SERCA2 and PLN was performed using 500 μg of each protein as described in Materials and Methods. (D) After HEK293T cells were co-transfected with human CFP-tagged PLN plasmids and YFP-tagged SERCA2a plasmids, cells were incubated for 30 min at 39°C using the heat block or for 30 min at RT or were exposed to FIR ray for 30 min at RT, and then the FRET values were measured as described in Materials and Methods. (E) VSMCs were incubated for 30 min at 39°C using the heat block, for 30 min at RT, or were exposed to FIR ray for 30 min at RT, and then intracellular ATP levels were measured using a luminescent ATP detection assay kit as described in Materials and Methods. All experiments were performed at least four times independently, and the confocal microscopic photographs and blots shown are representative of at least four experiments (n = 4). Bar graphs depict mean fold alterations above/below the controls (± SD). Differences were considered statistically significant at ^*^P < 0.05, ^**^P < 0.01, and ^***^P < 0.001.

Next, we explored whether these findings are attributable to the hyperthermal effect of FIR irradiation. As previously performed in our laboratory [26], the hyperthermal control was set to 39°C by placing culture dishes on the heat block for 30 min and further experiments were conducted. As shown in Fig 3A, the SER localization of intracellular Ca^2+^ was observed only under FIR-irradiated conditions, not hyperthermal conditions. The expression of SERCA2 and PLN were not altered in both FIR-irradiated and hyperthermal conditions (S4 Fig). However, the colocalization of SERCA2 and PLN was reduced only in FIR-irradiated VSMCs and, not in hyperthermia-treated cells (Fig 3B). In accordance with these qualitative results, the dissociation of SERCA2 and PLN was enhanced only under FIR-irradiated conditions, and not under hyperthermal conditions, as can be observed from the by co-IP and FRET assay results (Figs 3C and 3D). Furthermore, the intracellular ATP level was decreased only under FIR-irradiated conditions and, not under hyperthermal conditions (Fig 3E). These results showed that SER localization of intracellular Ca^2+^, promoted the dissociation of SERCA2 and PLN, and a decrease in intracellular ATP levels were caused by the distinctive effects of FIR, not hyperthermal effects.

### The reduction of cytosolic Ca^2+^ induced by FIR irradiation-activated SERCA2 suppresses p-MLC-Ser^19^ and VSMC contraction

**Fig 4:**
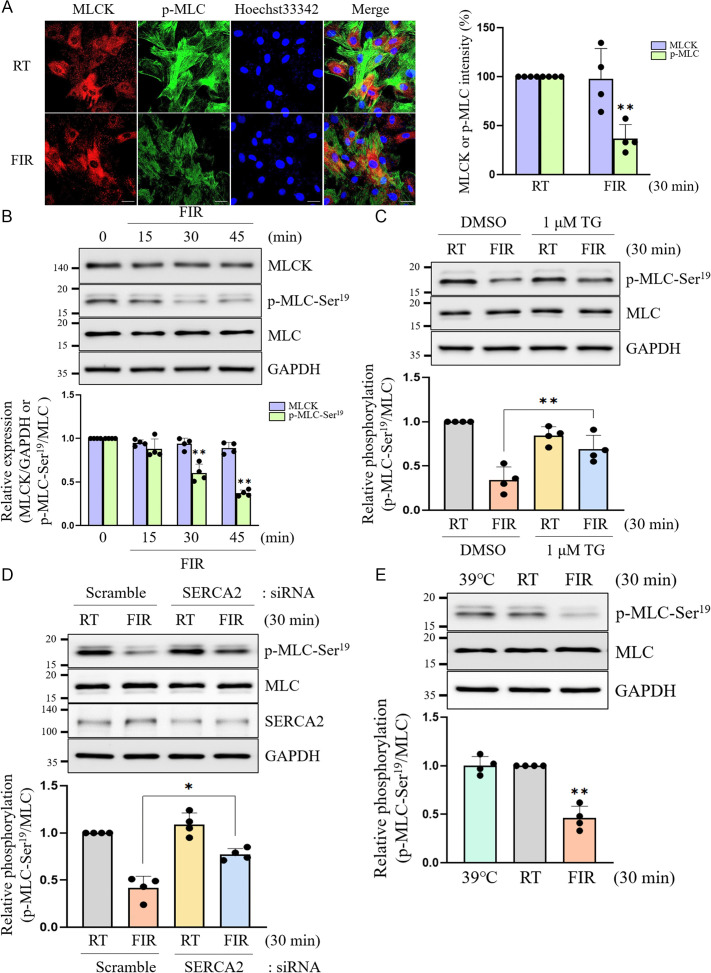
Depletion of cytosolic Ca^2+^ by FIR irradiation-activated SERCA2 results in the inhibition of p-MLC-Ser^19^ and VSMC contractility. (A) VSMCs grown on coverslips were irradiated with FIR ray or not for 30 min, and the presence of MLCK or p-MLC-Ser^19^ was detected by confocal microscopic analyses as described in Materials and Methods. The nuclei were also detected using 1 μM Hoechst33342. The scale bar indicates 30 µm. (B) After VSMCs were irradiated with FIR ray at indicated times (0, 15, 30, or 45 min), total proteins were obtained and the expression levels of MLCK, MLC, and p-MLC-Ser^19^ were assessed by western blotting as described in Materials and Methods. (C) VSMCs were incubated for 30 min in the absence or presence of 1 μM TG, irradiated with FIR ray or not for 30 min, and then the expression levels of p-MLC-Ser^19^ and MLC were detected by western blotting as described in Materials and Methods. (D) VSMCs were transfected with SERCA2-specific siRNA or scramble siRNA, followed by FIR irradiation for 30 min or not, and then the expression levels of p-MLC-Ser^19^, MLC, and SERCA2 were measured by western blotting as described in Materials and Methods. (E) VSMCs were incubated for 30 min at 39°C using the heat block or for 30 min at RT, or were exposed to FIR ray for 30 min at RT, and then the expression levels of p-MLC-Ser^19^ and MLC were assessed by western blotting as described in Materials and Methods. All experiments were performed at least four times independently, and the confocal microscopic photographs and blots shown are representative of at least four experiments (n = 4). Bar graphs depict mean fold alterations below the controls (± SD). Differences were considered statistically significant at ^*^P < 0.05 and ^**^P < 0.01.

Transient and sustained increases in sarcoplasmic Ca^2+^ concentrations are required to trigger and maintain muscle contraction of cardiac, skeletal, or smooth muscles [33,34]. Therefore, we investigated whether FIR irradiation decreases VSMC contractility, because our earlier results indicated that FIR irradiation lowered cytosolic Ca^2+^ through the SERCA2-mediated transport of intracellular Ca^2+^ into the SER (Fig 1E). As shown in Figs 4A and 4B, FIR irradiation significantly inhibited the phosphorylation of MLC at Ser19, with no alterations in the MLCK and MLC expressions. Furthermore, FIR irradiation-decreased p-MLC-Ser^19^ was significantly restored by pretreatment with TG or knockdown of SERCA2 gene expression (Figs 4C and 4D). As expected, FIR irradiation-decreased p-MLC-Ser^19^ was observed only under FIR-irradiated conditions, and not hyperthermal conditions (Fig 4E), suggesting that the decrease in p-MLC-Ser^19^ was due to the original effects of FIR rays, not hyperthermal effects. Collectively, these results demonstrated that FIR irradiation decreased p-MLC-Ser^19^ and consequent VSMC contractility by reducing cytosolic Ca^2+^ levels through the SERCA2-promoted transport of intracellular Ca^2+^ into the SER, independent of its hyperthermal effects.

### Activation of SERCA2 by FIR irradiation reduces PE-induced aortic vessel contraction and p-MLC-Ser^19^ in isolated rat aortas

**Fig 5:**
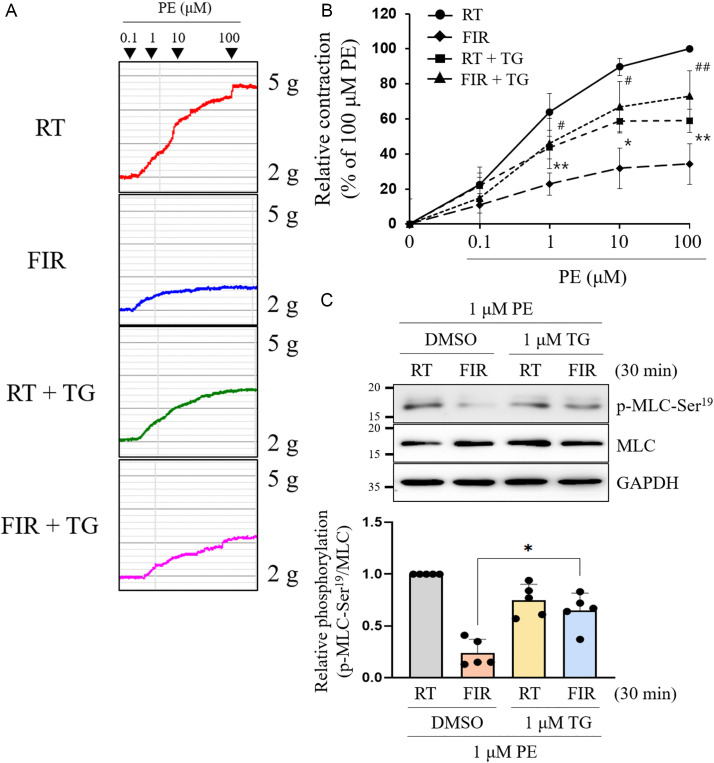
FIR irradiation attenuates p-MLC-Ser^19^ and PE-induced aortic vessel contraction by activating SERCA2. (A and B) Rat thoracic aortas were prepared and the vessel contraction assay was performed as described in Materials and Methods. After endothelium-deprived aortic rings were incubated for 1 h in the absence or presence of 1 μM TG and exposed to FIR ray or not for 30 min, PE-induced aortic contraction was measured by cumulative treatment with PE (0.1‒100 μM). The tension curves indicate PE-induced aortic contraction in response to each drug (A), and the line graph represents the mean ± SE at each point (n = 5) (B). (C) In a separate experiment, after endothelium-deprived aortic tissues were prepared as described above and treated with 1 μM PE for 10 min, total proteins were extracted and then p-MLC-Ser^19^ was assessed using western blotting as described in Materials and Methods. All experiments were independently performed at least five times, and the blots shown are representative of at least five experiments (n = 5). Bar graphs depict mean fold alterations below the controls (± SD). Differences were considered statistically signiﬁcant at ^*^P < 0.05, ^#^*P* < 0.05, ^**^P < 0.01, and ^##^*P* < 0.01.

To confirm whether the inhibitory effect of FIR irradiation on VSMC contraction, as well as its molecular mechanism identified from *in vitro* studies, are replicated in rat aortas, we conducted PE-induced vessel contraction assays and western blot analyses using isolated rat aortas. As shown in Figs 5A and 5B, pretreatment with 1 μM TG remarkably restored FIR irradiation-repressed aortic vessel contraction in endothelium-deprived rat aortas. Consistent with the *in vitro* findings, FIR irradiation-decreased p-MLC-Ser^19^ was significantly reversed in endothelium-deprived rat aortas pretreated with 1 μM TG (Fig 5C). These *ex vivo* findings indicate that FIR irradiation inhibits vessel contraction through a mechanism involving SERCA2-mediated reduction of MLC phosphorylation, suggesting its potential relevance *in vitro*, *ex vivo*, and possibly *in vivo*, thus linking to physiological function (Fig 7).

**Fig 6:**
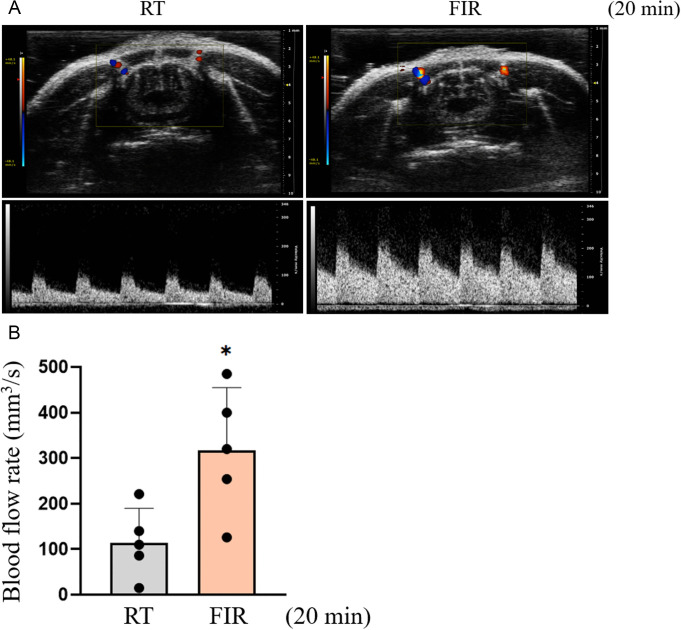
FIR irradiation enhances the blood flow rate in the carotid artery of mice. (A and B) Peripheral blood flow to the skin vasculature was assessed by measuring the blood flow rate in the carotid artery of mice using ultrasound imaging analysis as described in Materials and Methods. Vessel diameter and blood flow velocity were measured in the carotid artery of mice using color Doppler mode and pulsed-wave Doppler mode, respectively (A). The blood flow rate was calculated by multiplying the vessel diameter by the mean blood flow velocity (B). All experiments were independently performed at least five times, and the ultrasound images shown are representative of at least five experiments (n = 5). Bar graphs depict mean fold alterations above the controls (± SD). Differences were considered statistically signiﬁcant at ^*^P < 0.05.

**Fig 7:**
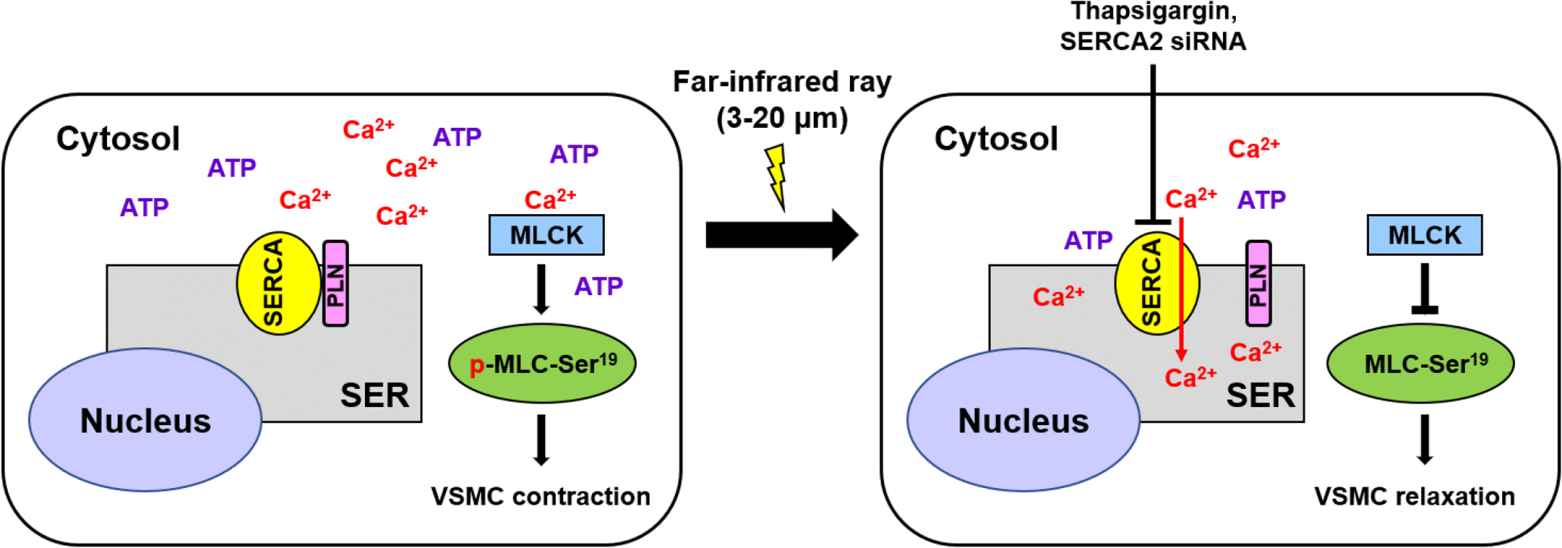
A schematic illustration of FIR irradiation-inhibited VSMC contraction. FIR irradiation activates SERCA2 by promoting the dissociation of SERCA2 and PLN irrespective of the hyperthermal effect of FIR rays. FIR irradiation-activated SERCA2 enhances the transport of cytosolic Ca^2+^ into the SER at the expense of ATP consumption. Finally, diminished cytosolic Ca^2+^ and ATP decrease VSMC contraction by inhibiting MLC phosphorylation.

### FIR irradiation increases the blood flow rate in the carotid artery of mice

Finally, we examined whether FIR irradiation increases the blood flow rate in the carotid artery of mice using ultrasound imaging analysis, which reflects peripheral blood flow to the skin vasculature. As shown in Figs 6A and 6B, the blood flow rate in the carotid artery of FIR-irradiated mice increased 3-fold compared to that of control mice. These results demonstrate that the vasorelaxation effects of FIR irradiation observed *in vitro* and *ex vivo* indeed promoted peripheral blood flow to the skin vasculature *in vivo*.

## Discussion

Dysregulated contractility of VSMCs is recognized as a key factor in the pathogenesis of several vascular diseases, including pathological vasospasms [5]. One example of this is coronary artery spasm, which plays a crucial role in the development of ischemic heart diseases, including angina pectoris, myocardial infarction, and sudden cardiac death [35]. Similarly, post-SAH cerebral vasospasm, which significantly contributes to patient morbidity and mortality, is underpinned by an initial surge in VSMC contractility, followed by a phase of persistent yet reversible vasoconstriction of the cerebral arteries. [6]. The mobilization of stored Ca^2+^ from the SER into the cytosol through the binding of inositol 1,4,5‐trisphosphate (IP_3_) to IP_3_ receptor, a Ca^2+^ channel, on the SER membrane give rise to excessive sarcoplasmic Ca^2+^ levels in VSMCs, which results in VSMC hypercontraction [5]. Furthermore, estrogen increases the expression level of SERCA2b in female porcine coronary arteries, which restricts the excessive accumulation of sarcoplasmic Ca^2+^ and potentially coronary vasospasms [36]. In line with these reports, our current results revealed that FIR irradiation inhibits VSMC contraction by decreasing cytosolic Ca²⁺ and ATP levels, leading to reduced MLC phosphorylation via SERCA2 activation (Fig 7). These observations imply that FIR therapy could be a potential therapeutic option for pathological vasospasms, including coronary artery spasm and post-SAH vasospasm.

An important finding in this study is that FIR irradiation elevated the activity of SERCA2 by promoting the dissociation of SERCA2 and PLN, resulting in a reduction of Ca^2+^ and ATP levels in the cytoplasm (Figs 1 and 2). These results directly suggest that SERCA2 is the biological mediator of FIR ray. The energy of an electromagnetic wave decreases as its wavelength increases, and therefore, electromagnetic waves with different wavelengths exert their biological effects through a specialized cellular mediator that can perceive the range of wavelength of electromagnetic wave with specific energy levels in living organisms [37–40]. The photon energy level at the 500 nm wavelength range within visible light (VIS) is 2.48 eV, and the energy at this level can give rise to the photochemical isomerization of the double bond of 11-*cis*-retinal to the 11-*trans*-cofiguration and subsequently rhodopsin conformation, ultimately generating vision signal in the human retina [38]. Thus, the G-protein coupled receptor rhodopsin containing 11-*cis*-retinal is the biological mediator of VIS. The photon energy level of infrared (IR) ranging from the 0.75 to 1 µm wavelength is 1.65 to 1.24 eV, and the energy at these levels is perceived as a form of heat by venomous pit vipers through the transient receptor potential (TRP) vanilloid 1 Ca^2+^ channel expressed in the pit organ of the snakes [39]. Furthermore, vampire bats perceive the IR energy through the short form of the alternative splicing variant of the TRP ankyrin 1 Ca^2+^ channel [40]. In these cases, photon energy of IR causes the conformational change of specific Ca^2+^ channel. Thus, these channels are the biological mediator of IR in some vipers and vampire bats. On the other hand, FIR rays have a photon energy range of 0.41 eV to 12.4 meV which is considerably lower than that of other IR rays. [19]. The photon energy at these levels are unlikely to cause alterations in the isomerization of double bonds in a photochromophore and/or protein conformation. Instead, the photon energy of FIR rays is likely to change a protein-protein interaction and membrane fluidity, because FIR irradiation can modify the vibrational characteristics of bonds in molecules like water, proteins, and lipids by influencing multiple vibrational modes such as stretching, scissoring, and twisting [19,41]. Furthermore, FIR rays can induce the meso-structural effect, which alters the dielectric properties of the entire molecular assembly [19]. In this respect, the photon energy of FIR rays are thought to disrupt the SERCA2 and PLN complex by increasing vibrations of the two proteins, because PLN, an endogenous and small integral membrane protein comprising 52 amino acids, binds to SERCA2 through weak intermolecular interactions like Van der Waals forces and hydrogen bonds [13,16,17]. Based on these facts and the current results showing that FIR irradiation activated SERCA2 by diminishing the intermolecular interaction of SERCA2 and PLN (Figs 1 and 2), SERCA2 is likely the biological mediator of FIR rays.

Reduced expression and activity of SERCA are linked to the onset of several cardiovascular diseases, including heart failure [42,43], and SERCA activation by istaroxime promoting Ca^2+^ re-uptake into the SER via SERCA2a has been recently reported to improve diastolic dysfunction in streptozotocin-induced cardiomyopathy model rats [44]. In addition, the stimulation of SERCA2b activity using CDN1163 attenuates diabetes and metabolic disorders by reducing ER stress in ob/ob mice [45]. In the current study, our results obviously revealed that FIR irradiation increased SERCA activity in rat VSMCs (Fig 1B), which translocated intracellular Ca^2+^ into the SER (Fig 1E). Considering these reports and our current results, FIR therapy as a non-pharmacologic intervention that can activates SERCA in the body may be useful for the prevention and treatment of cardiovascular and metabolic diseases. In supporting this notion, the repeated FIR sauna therapy improves clinical signs and symptoms of congestive heart failure in patients with chronic systolic heart failure [46], and significantly lowers body fat mass and body weight in obese patients [47].

Recently, it has been documented that in breast cancer cells, FIR irradiation under conditions identical to those in our current study resulted in the translocation of intracellular Ca^2+^ into the nucleus [26]. Similarly, consistent with this finding, exposure of VSMCs to FIR ray resulted in a shift of intracellular Ca^2+^ localization from the cytosol to the SER (Fig 1A). In addition, our current results showed that FIR irradiation significantly depleted cellular ATP levels during this Ca^2+^ transport through FIR irradiation-activated SERCA (Fig 1C). In this respect, it is reported that a significant quantity of ATP is utilized to transport ions across cell membranes against their concentration gradients [48], and SERCA uses one molecule of ATP to transport two cytosolic Ca^2+^ to the SER against the concentration gradient [13,14]. In resting conditions, skeletal muscles utilize 40–50% of their total cellular ATP to transport Ca^2+^ from the cytosol to the SER [49]. Furthermore, the uncoupling of SERCA and its endogenous small peptide inhibitor including SLN can give rise to heat generation by consuming ATP in skeletal muscle and beige fat without concomitant Ca^2+^ transport through so-called futile Ca^2+^ cycling [50]. Based on our current results and these reports, FIR therapy may be applied for the prevention and treatment of diverse metabolic diseases, particularly diabetes and obesity. If so, in the future, the findings from this study provide a scientific foundation for using FIR therapy in the prevention and treatment of diabetes and obesity.

Hyperthermal condition (39°C) did not alter intracellular Ca^2+^ mobilization, the binding status of SERCA2 and PLN, and intracellular ATP level, indicating that the FIR irradiation-induced SER localization of Ca^2+^, promoted dissociation of SERCA2 and PLN, and depletion of intracellular ATP originated from the peculiar effect of FIR, not its hyperthermal effect (Fig 3). In supporting these results, FIR irradiation enhances skin wound healing in rats without altering skin temperature before or during FIR exposure [51]. In addition, FIR irradiation increases the skin microcirculation in SD rats [52] irrespective of hyperthermia, and decreases the migration and angiogenesis of human endothelial cells through non-thermal biological effects [25]. Based on the findings from this study and numerous previous reports, it is probable that FIR rays exert their biological effects through a pathway that is not primarily based on heat. Nonetheless, further structural and functional studies on numerous TRP channels in the presence of FIR rays are needed to clarify this issue, because a wide range of TRPs can sense different ranges of temperature in the body [53].

The findings from *ex vivo* experiments provide compelling evidence that FIR irradiation decreased the activation of intracellular signaling pathways triggered by vasoconstrictive agents including PE (Fig 5C). Various medical conditions such as hormonal imbalances, renal insufficiency, aging, and metabolic disorders have been shown to intensify the signaling of vasoconstrictors, resulting in dysfunctions of VSMCs. [54]. For example, in spontaneously hypertensive rats, angiotensin II-induced stiffness in VSMCs and subsequent stiffness in the aorta are heightened, which is associated with increased expression of MLCK and phosphorylation of MLC [55]. In addition, passive aortic stiffness and PE-induced aortic stiffness are more severe in old mouse aortas compared to those in young control aortas, with VSMCs playing a significant role in contributing to this aortic stiffness [56]. Based on the findings from these studies and the current results demonstrating that FIR irradiation reduced PE-induced contraction of aortic vessels (Fig 5), FIR irradiation is anticipated to ameliorate vascular diseases associated with arterial stiffness and aging, including stroke and arteriosclerosis, by reducing vasoconstrictor signaling.

## Conclusion

Our current results revealed that FIR irradiation increases SERCA2 activity by promoting the dissociation of SERCA2 and PLN irrespective of its hyperthermal effect, which decreases VSMC contraction by diminishing cytosolic Ca^2+^ and ATP levels, consequently MLC phosphorylation (Fig 7). These novel findings offer scientific evidence suggesting that FIR therapy, as a non-invasive approach, could be beneficial for both preventing and treating conditions characterized by arterial narrowing, such as atherosclerosis and in-stent restenosis. Additionally, it may be effective in addressing pathological vasospasms, such as coronary artery spasm and post-SAH vasospasm.

## Supporting information

S1 File Supplementary Figure. (PDF)

S1 Fig Intracellular Ca^2+^ is localized to the perinucleus by FIR irradiation. VSMCs grown on coverslips were irradiated with FIR ray or not for 30 min and treated with Fura-2AM. Intracellular Ca^2+^ locations were detected by confocal microscopic analyses as described in Materials and Methods. The nuclei were also detected by treatment with Hoechst33342. The experiments were performed at least four times independently, and the confocal microscopic photographs shown are representative of at least four experiments (n = 4). The scale bar indicates 30 µm.
(TIF)

S2 Fig SERCA2 gene expression was successfully knockdown in *Atp2a2* siRNA- transfected VSMCs. VSMCs were transfected with SERCA2-specific siRNA or scramble siRNA, followed by FIR or not for 30 min. Total proteins were obtained and the expression levels of SERCA2 were assessed by western blotting as described in Materials and Methods. The experiments were performed at least four times independently (n = 4). Bar graphs depict mean fold alterations below the controls (± SD). Differences were considered statistically significant at ^*^P < 0.05.
(TIF)

S3 Fig The expression levels of SERCA2 and PLN are not altered in FIR-irradiated VSMCs. After VSMCs were irradiated with FIR ray for indicated times (0, 15, 30, or 45 min), total proteins were obtained and the expression levels of SERCA2 and PLN were evaluated by western blotting as described in Materials and Methods. All experiments were performed at least four times independently and the blots shown are representative of at least four experiments (n = 4). The bar graph depicts mean fold alterations above/below the controls (± SD).
(TIF)

S4 Fig The expression levels of SERCA2 and PLN are not changed in both FIR- irradiated and hyperthermal conditions. VSMCs were incubated for 30 min at 39°C using the heat block or for 30 min at RT or were exposed to FIR ray for 30 min at RT, and then the expression levels of SERCA2 and PLN were detected by western blotting as described in Materials and Methods. The experiments were performed at least four times independently, and the blots shown are representative of at least four experiments (n = 4). Bar graphs depict mean fold alterations above/below the controls (± SD).
(TIF)

S2 File Raw images. Original images of blot for Fig 2–5 and Supplementary Fig 2–4.
(PDF)
