# Supplementary figures and images for: Far-infrared irradiation attenuates vessel contraction by activating SERCA2 through disruption of SERCA2 and PLN interaction
Source: PLoS One. 2025 Dec 17;20(12):e0339066. doi: 10.1371/journal.pone.0339066 (PMC12711061; doi:10.1371/journal.pone.0339066)

Supplementary Fig. S1

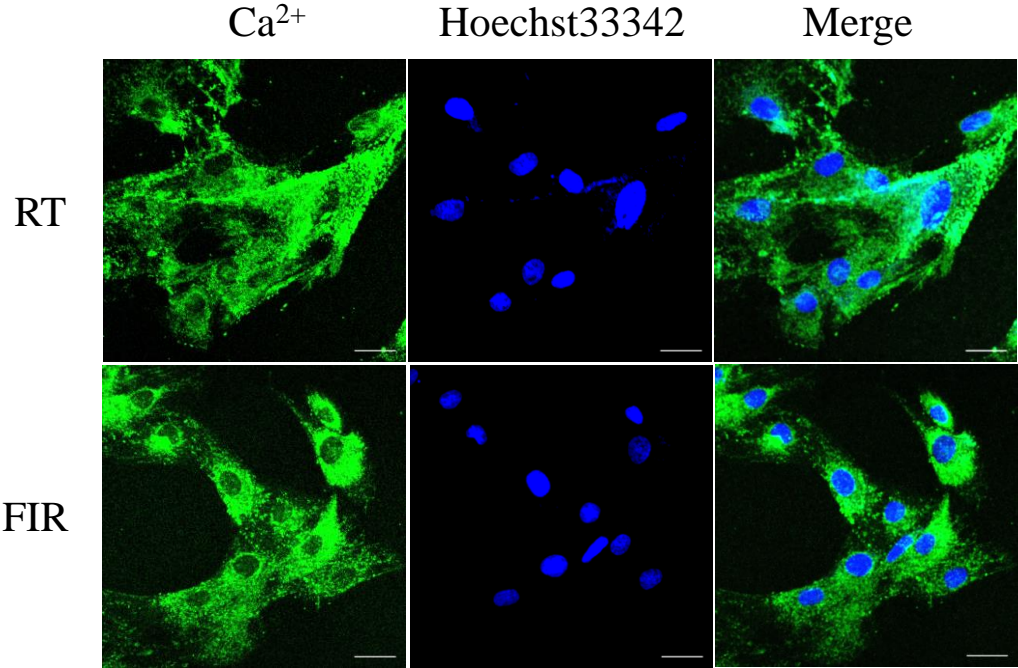

Supplementary Fig. S2

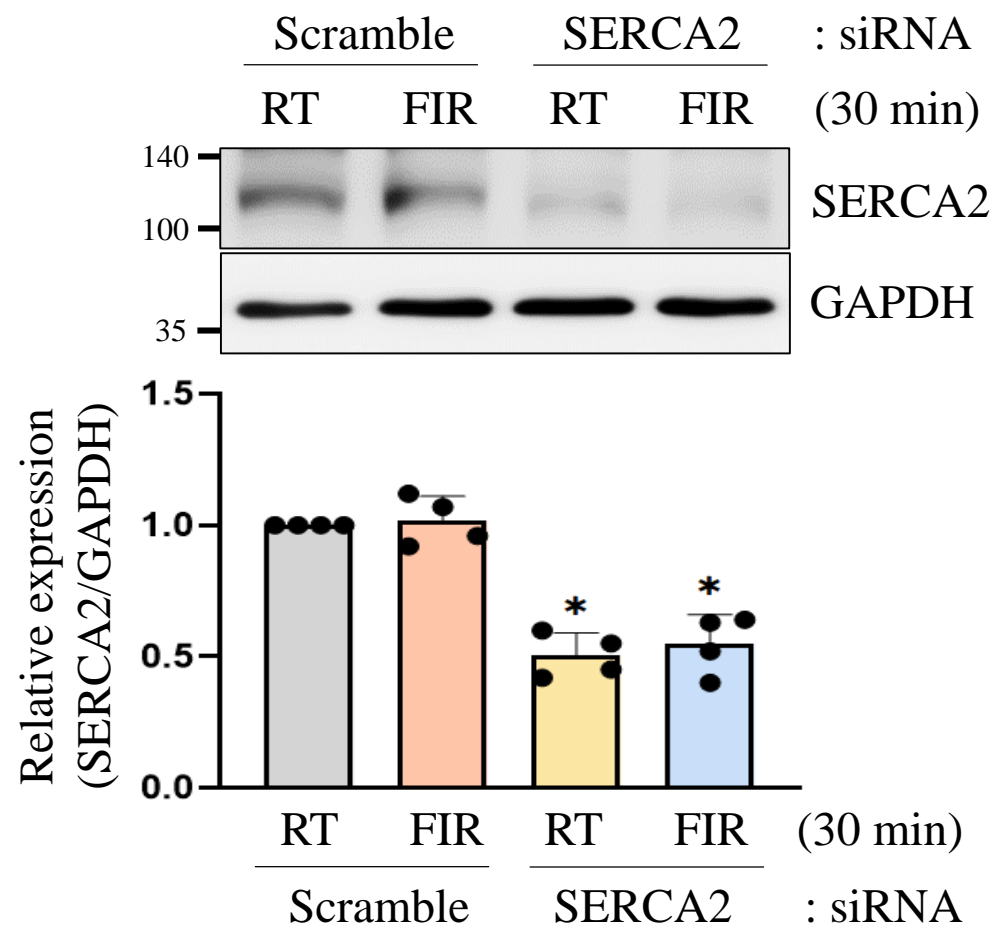

Supplementary Fig. S3

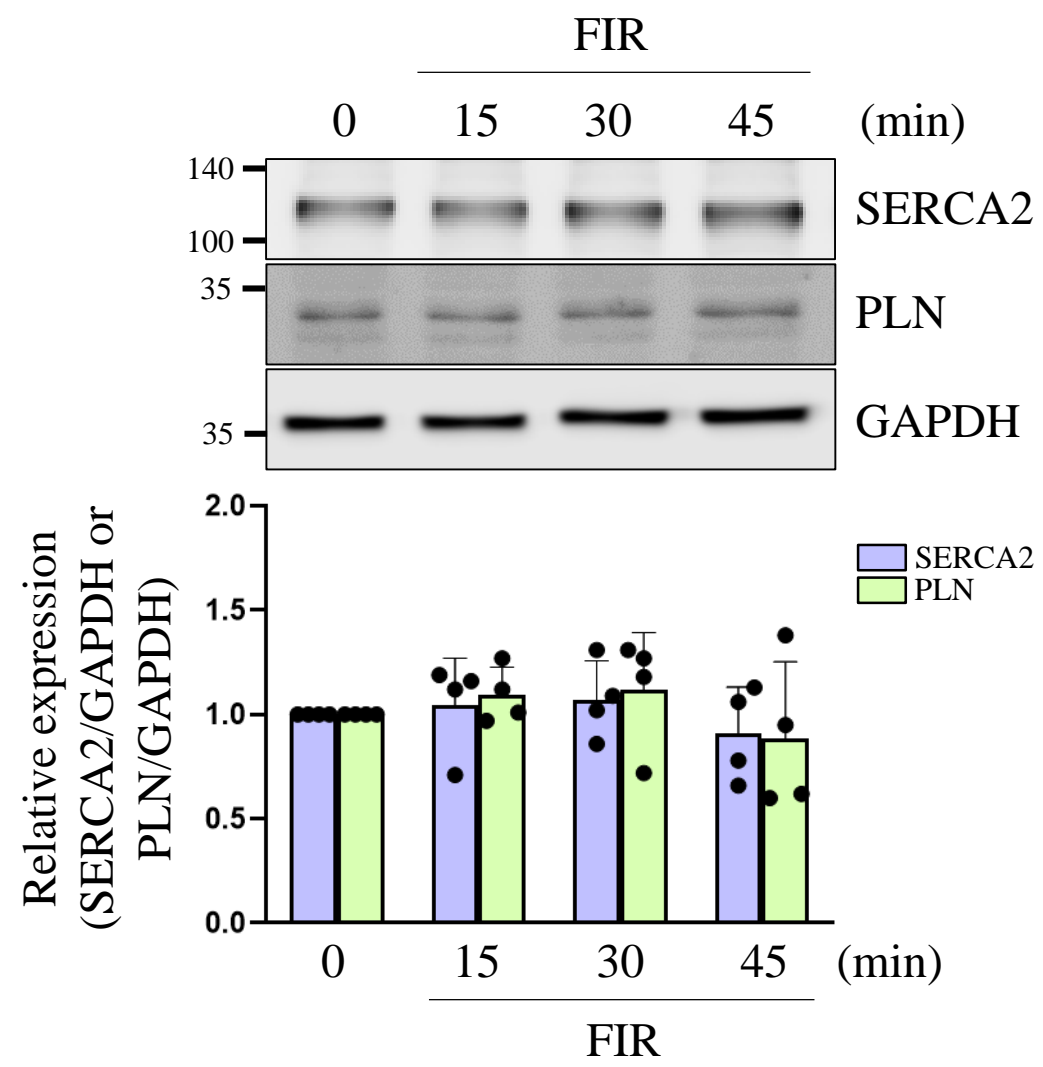

Supplementary Fig. S4

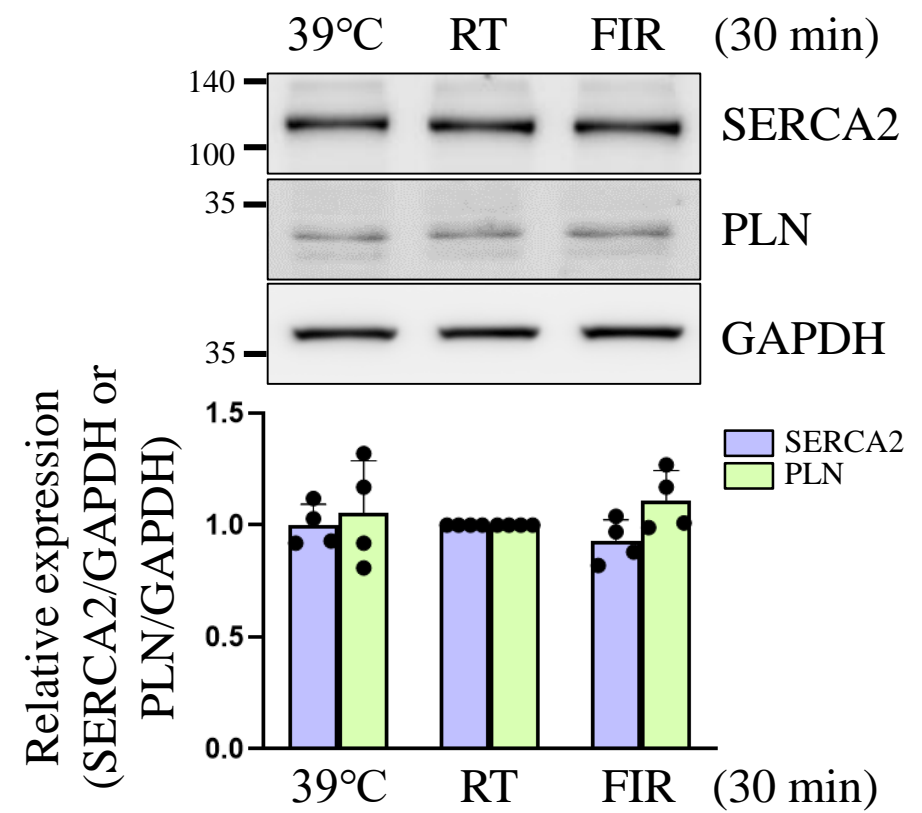

Supplement: S1 File — (PDF) [file pone.0339066.s001.pdf]
