# Supplementary material for: Far-infrared irradiation attenuates vessel contraction by activating SERCA2 through disruption of SERCA2 and PLN interaction
Source: PLoS One. 2025 Dec 17;20(12):e0339066. doi: 10.1371/journal.pone.0339066 (PMC12711061; doi:10.1371/journal.pone.0339066)
Supplement: S2 File — Original images of blot for Fig 2–5 and Supplementary Fig 2–4. (PDF) [file pone.0339066.s006.pdf]

Fig. 2B

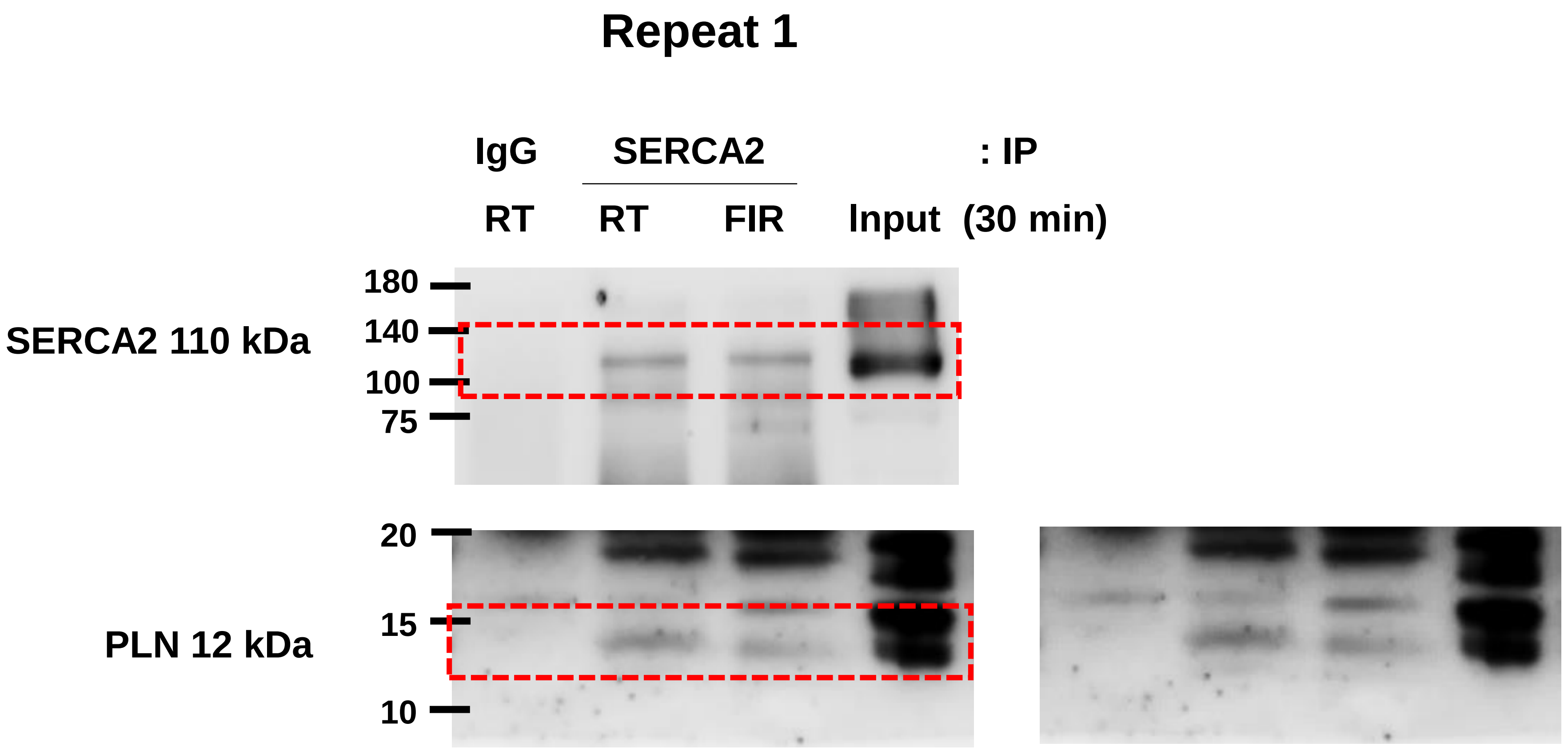
Repeat 1
IgG
SERCA2
: IP
RT
RT
FIR
Input
(30 min)
SERCA2 110 kDa
180
140
100
75
PLN 12 kDa
20
15
10

Fig. 2B

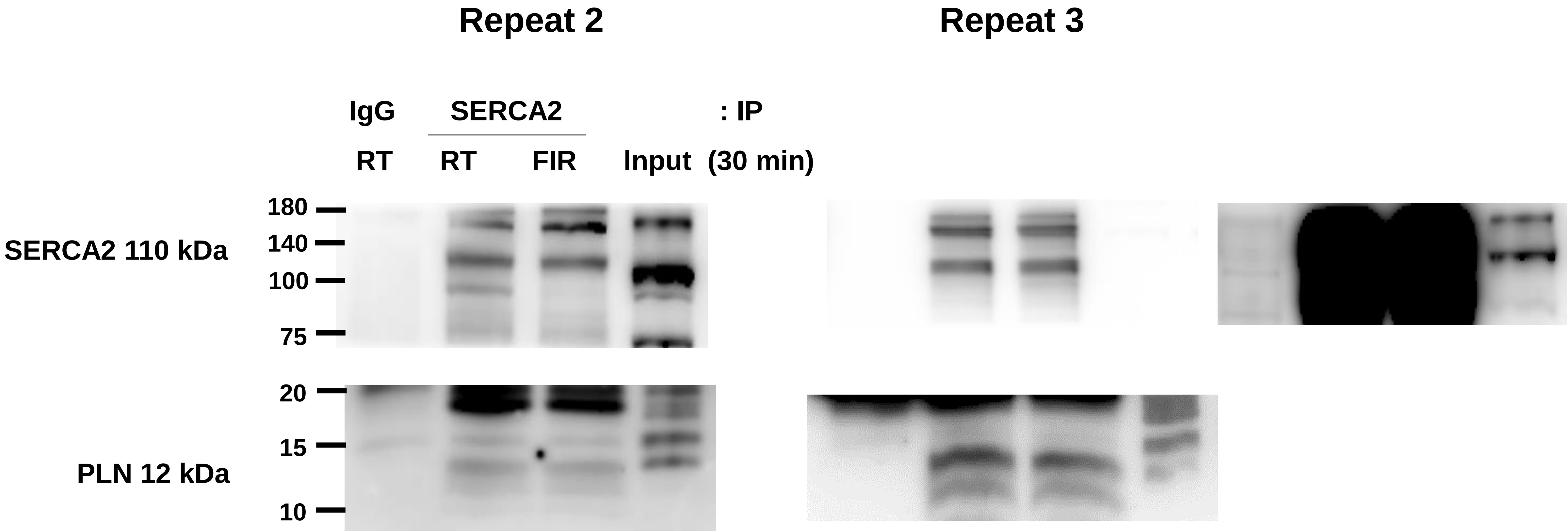

Repeat 2
Repeat 3
IgG
SERCA2
: IP
RT
RT
FIR
Input
(30 min)
180
140
100
75
SERCA2 110 kDa
20
15
10
PLN 12 kDa

Fig. 3C

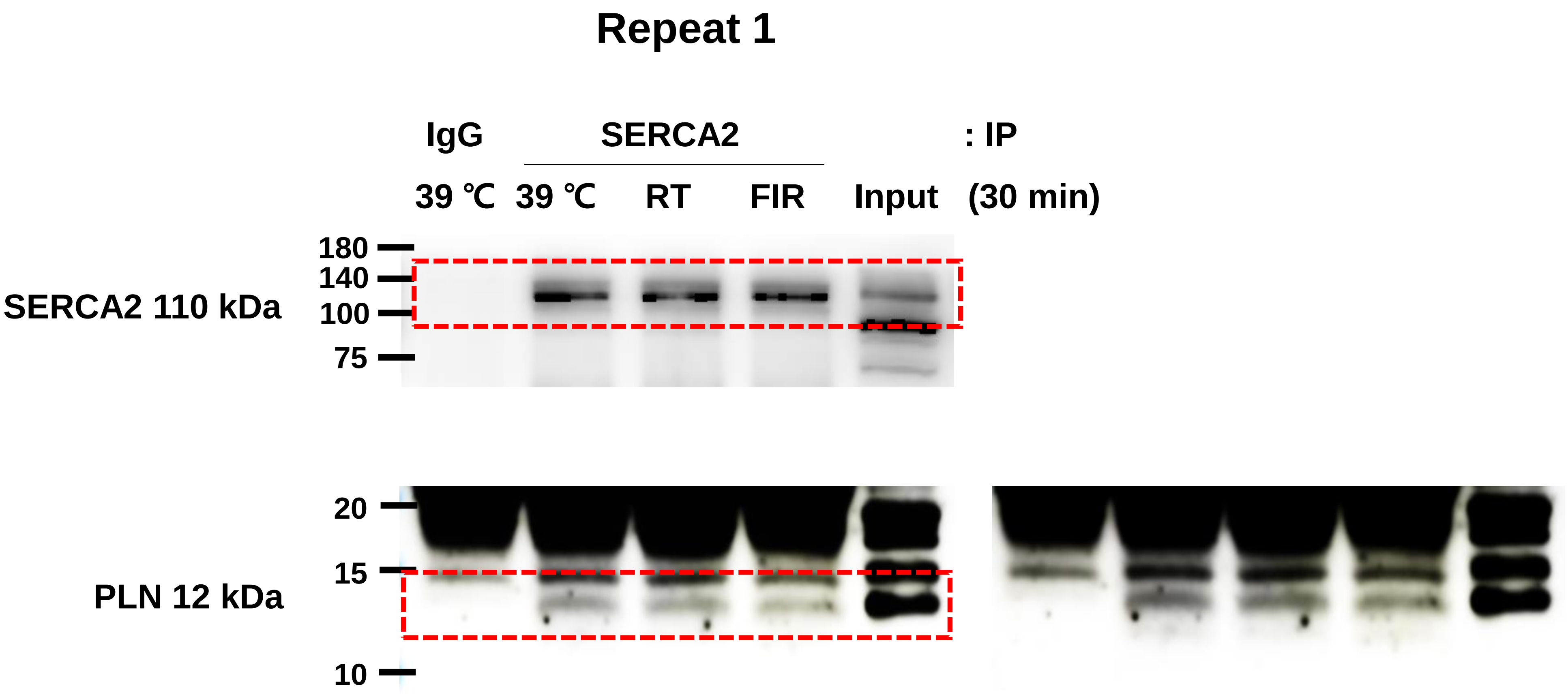
Repeat 1
IgG
SERCA2
: IP
39 ℃
39 ℃
RT
FIR
Input
(30 min)
SERCA2 110 kDa
180
140
100
75
PLN 12 kDa
20
15
10

Fig. 3C

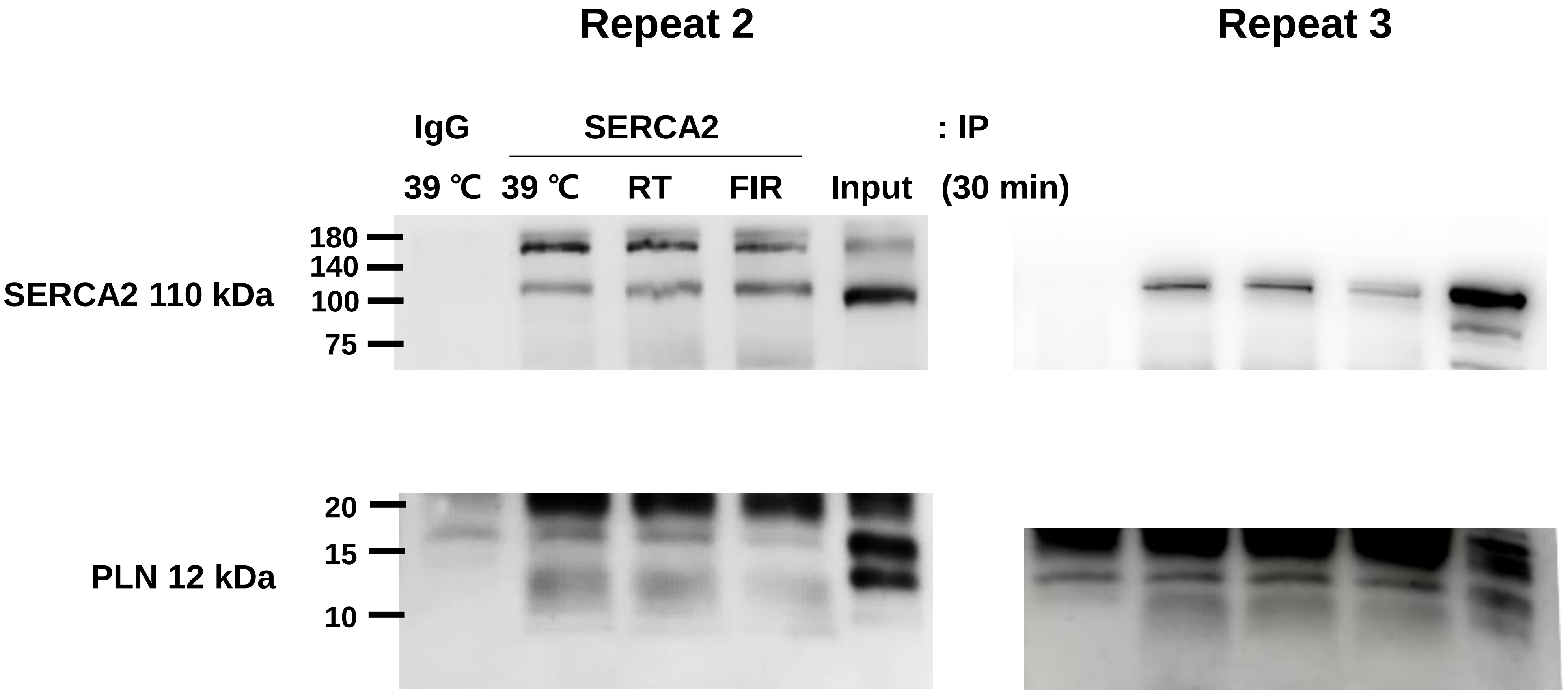
Repeat 2
Repeat 3
IgG
SERCA2
: IP
39 ℃
39 ℃
RT
FIR
Input
(30 min)
180
140
100
75
SERCA2 110 kDa
20
15
10
PLN 12 kDa

Fig. 4B

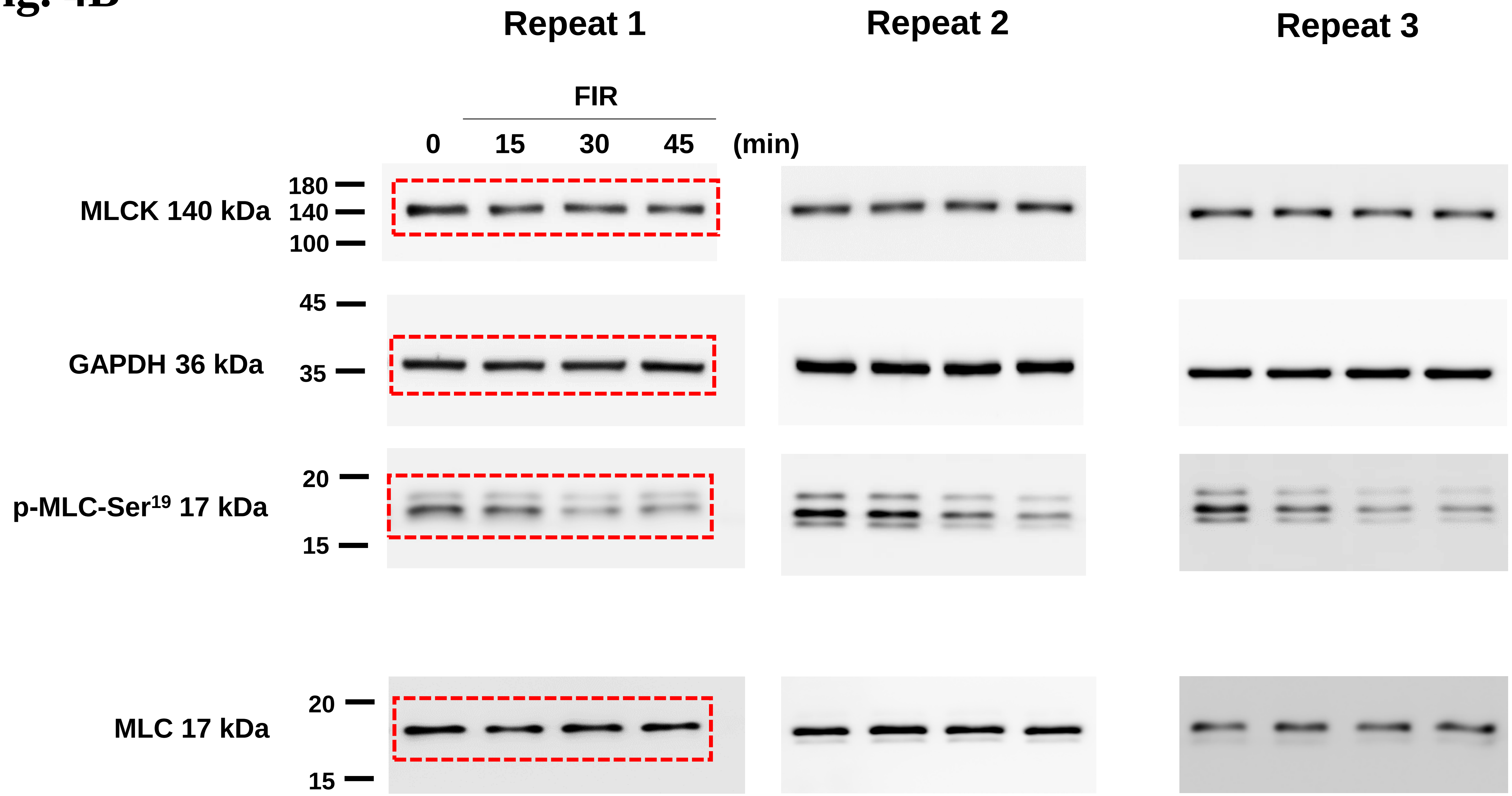

Repeat 1
Repeat 2
Repeat 3
FIR
0
15
30
45
(min)
MLCK 140 kDa
180
140
100
45
GAPDH 36 kDa
35
20
p-MLC-Ser19 17 kDa
15
20
MLC 17 kDa
15

Fig. 4C

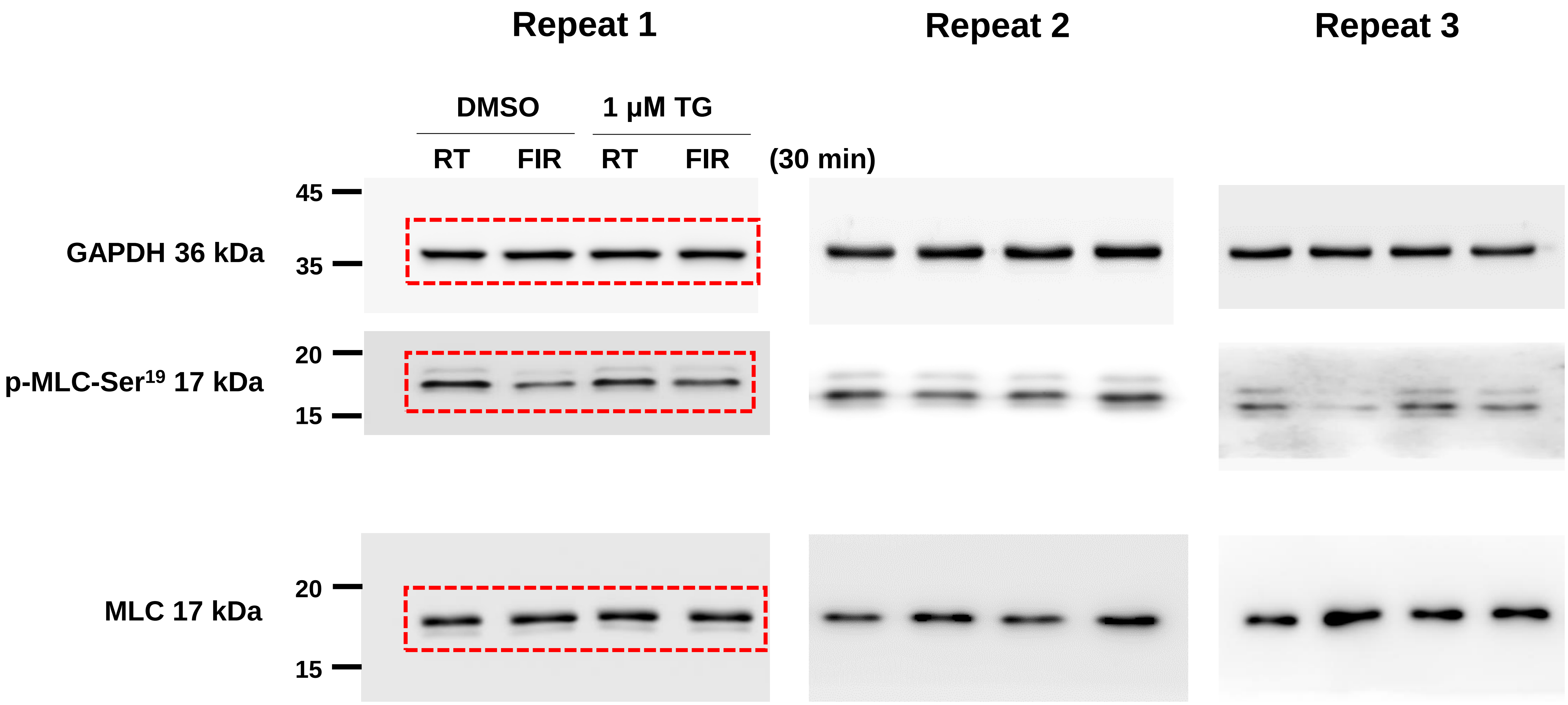
Repeat 1
Repeat 2
Repeat 3
DMSO
1 μM TG
RT
FIR
RT
FIR
(30 min)
GAPDH 36 kDa
p-MLC-Ser19 17 kDa
MLC 17 kDa
45
35
20
15
20
15

Fig. 4D

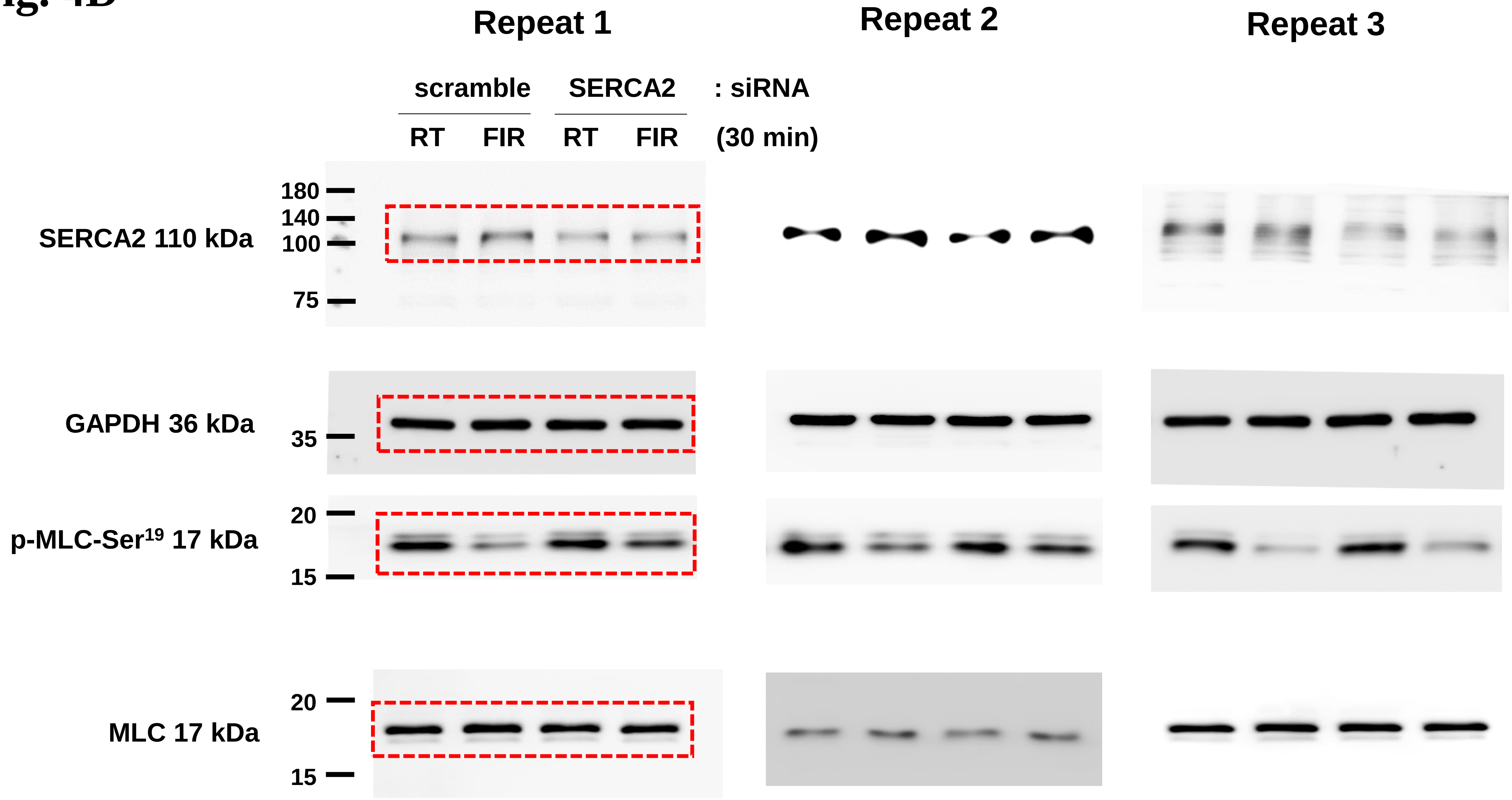
Repeat 1
Repeat 2
Repeat 3
scramble
SERCA2
: siRNA
RT
FIR
RT
FIR
(30 min)
SERCA2 110 kDa
180
140
100
75
GAPDH 36 kDa
35
p-MLC-Ser19 17 kDa
20
15
MLC 17 kDa
20
15

Fig. 4E

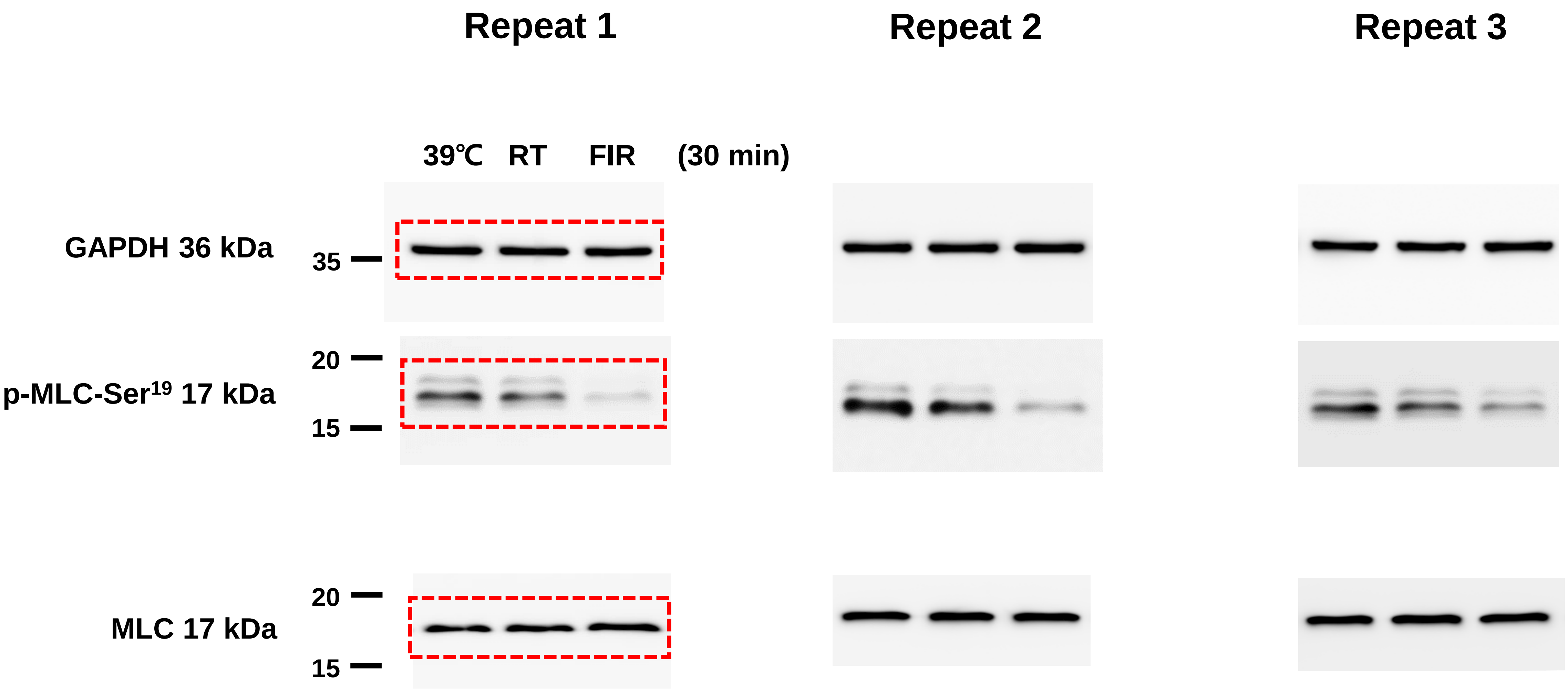
Repeat 1
Repeat 2
Repeat 3
39℃
RT
FIR
(30 min)
GAPDH 36 kDa
35
20
p-MLC-Ser19 17 kDa
15
20
MLC 17 kDa
15

Fig. 5C

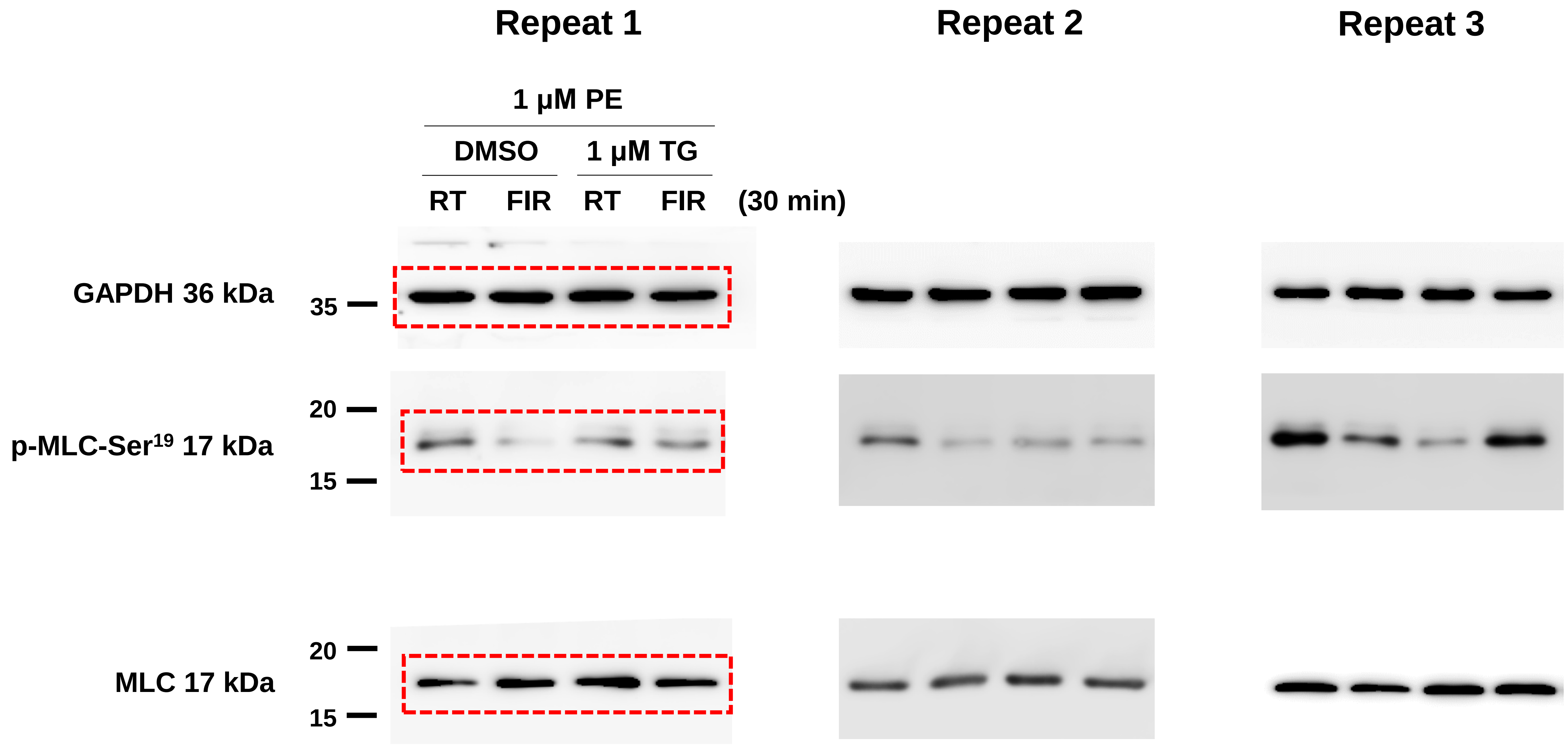

Repeat 1
Repeat 2
Repeat 3
1 μM PE
DMSO
1 μM TG
RT
FIR
RT
FIR
(30 min)
GAPDH 36 kDa
35
20
p-MLC-Ser19 17 kDa
15
20
MLC 17 kDa
15

S2 Fig.

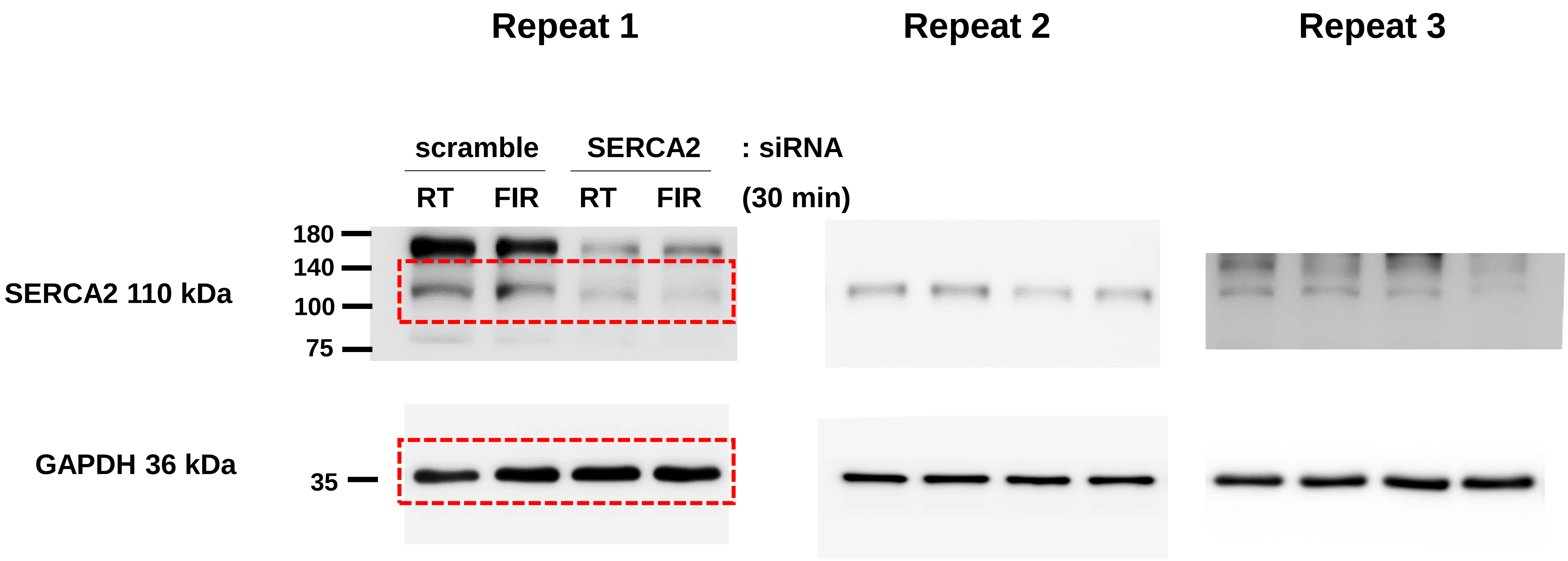

Repeat 1
Repeat 2
Repeat 3
scramble
SERCA2
: siRNA
RT
FIR
RT
FIR
(30 min)
180
140
100
75
SERCA2 110 kDa
GAPDH 36 kDa
35

S3 Fig.

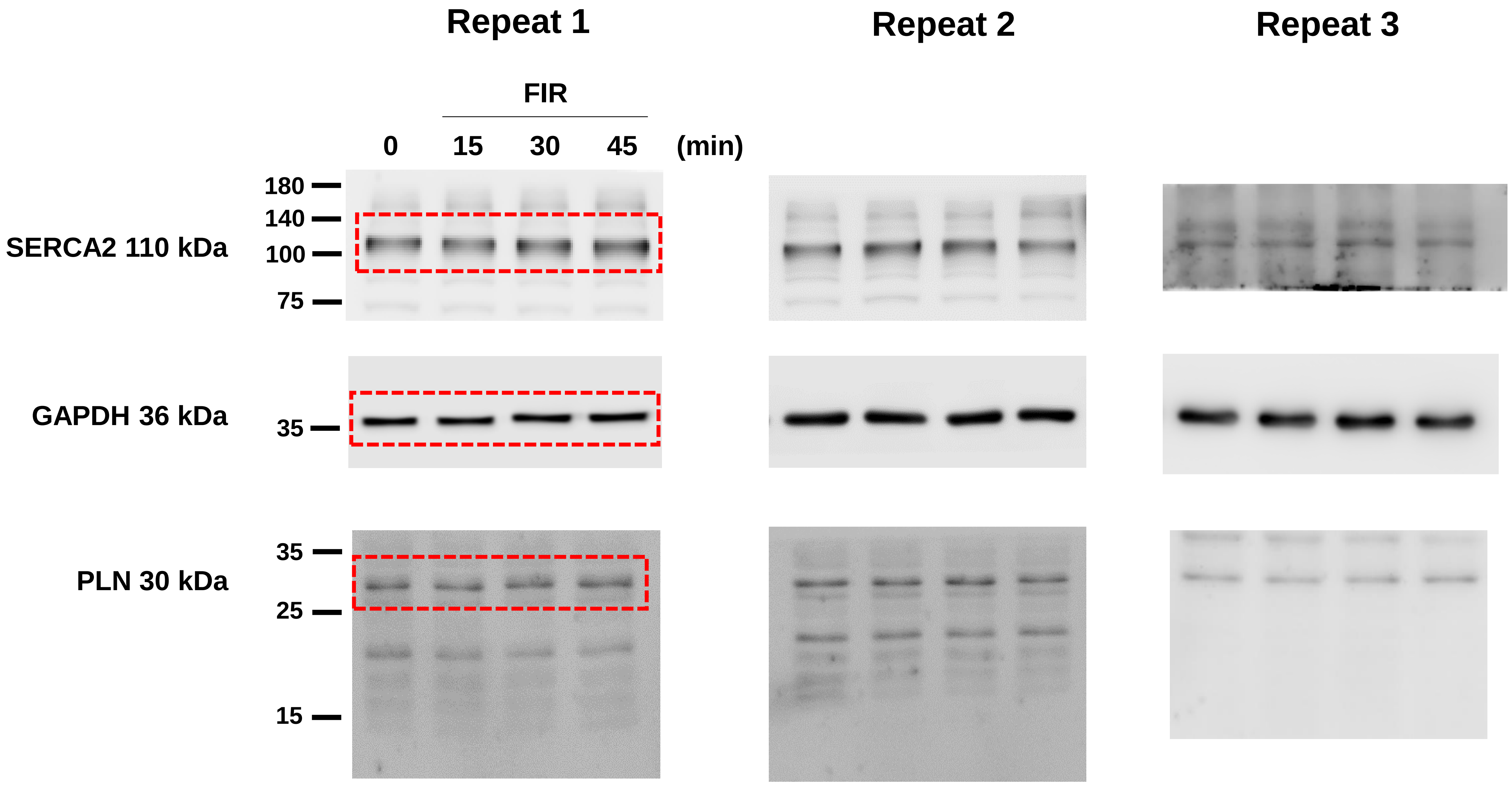

Repeat 1
Repeat 2
Repeat 3
FIR
0
15
30
45
(min)
SERCA2 110 kDa
180
140
100
75
GAPDH 36 kDa
35
PLN 30 kDa
35
25
15

S4 Fig.

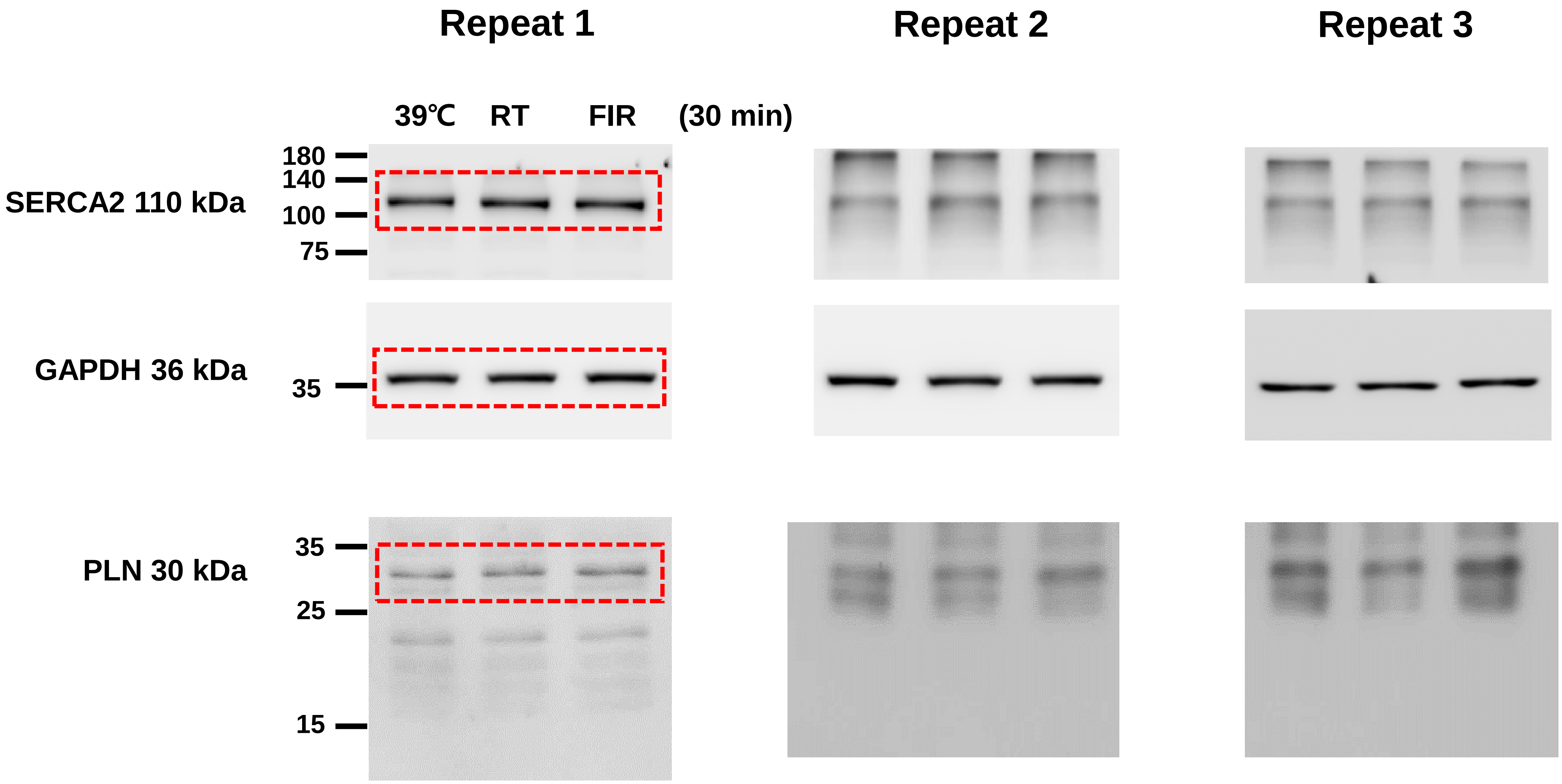

Repeat 1
Repeat 2
Repeat 3
39℃
RT
FIR
(30 min)
SERCA2 110 kDa
GAPDH 36 kDa
PLN 30 kDa
180
140
100
75
35
35
25
15
